# Structure-Guided Design of a Fluorescent Probe for the Visualization of FtsZ in Clinically Important Gram-Positive and Gram-Negative Bacterial Pathogens

**DOI:** 10.1038/s41598-019-56557-x

**Published:** 2019-12-27

**Authors:** Edgar Ferrer-González, Junso Fujita, Takuya Yoshizawa, Julia M. Nelson, Alyssa J. Pilch, Elani Hillman, Mayuki Ozawa, Natsuko Kuroda, Hassan M. Al-Tameemi, Jeffrey M. Boyd, Edmond J. LaVoie, Hiroyoshi Matsumura, Daniel S. Pilch

**Affiliations:** 1 Department of Pharmacology, Rutgers Robert Wood Johnson Medical School, 675 Hoes Lane, Piscataway, NJ 08854 USA; 2 Department of Applied Chemistry, Graduate School of Engineering, Osaka University, 2-1 Yamadaoka, Suita, Osaka 565-087 Japan; 3 Department of Biotechnology, College of Life Sciences, Ritsumeikan University, 1-1-1 Noji-Higashi, Shiga, 525-8577 Japan; 4 Department of Biochemistry and Microbiology, School of Environmental and Biological Sciences, Rutgers University, 76 Lipman Drive, New Brunswick, NJ 08901 USA; 5 Department of Medicinal Chemistry, Ernest Mario School of Pharmacy, Rutgers University, 160 Frelinghuysen Road, Piscataway, NJ 08854 USA; 6 Present Address: MRC Laboratory of Molecular Biology, Francis Crick Avenue, Cambridge Biomedical Campus, Cambridge, CB2 0QH UK

**Keywords:** Antibiotics, Phenotypic screening

## Abstract

Addressing the growing problem of antibiotic resistance requires the development of new drugs with novel antibacterial targets. FtsZ has been identified as an appealing new target for antibacterial agents. Here, we describe the structure-guided design of a new fluorescent probe (**BOFP**) in which a BODIPY fluorophore has been conjugated to an oxazole-benzamide FtsZ inhibitor. Crystallographic studies have enabled us to identify the optimal position for tethering the fluorophore that facilitates the high-affinity FtsZ binding of **BOFP**. Fluorescence anisotropy studies demonstrate that **BOFP** binds the FtsZ proteins from the Gram-positive pathogens *Staphylococcus aureus*, *Enterococcus faecalis*, *Enterococcus faecium*, *Streptococcus pyogenes*, *Streptococcus agalactiae*, and *Streptococcus pneumoniae* with K_d_ values of 0.6–4.6 µM. Significantly, **BOFP** binds the FtsZ proteins from the Gram-negative pathogens *Escherichia coli*, *Klebsiella pneumoniae*, *Pseudomonas aeruginosa*, and *Acinetobacter baumannii* with an even higher affinity (K_d_ = 0.2–0.8 µM). Fluorescence microscopy studies reveal that **BOFP** can effectively label FtsZ in all the above Gram-positive and Gram-negative pathogens. In addition, **BOFP** is effective at monitoring the impact of non-fluorescent inhibitors on FtsZ localization in these target pathogens. Viewed as a whole, our results highlight the utility of **BOFP** as a powerful tool for identifying new broad-spectrum FtsZ inhibitors and understanding their mechanisms of action.

## Introduction

The discovery and development of antibiotics have saved millions of lives and revolutionized modern medicine^1^. However, the alarming rise in multidrug-resistant (MDR) bacteria has threatened the usefulness of our current arsenal of antibiotics, leading the World Health Organization (WHO) to suggest an impending post-antibiotic era, where minor infections would become lethal^2^. A recent WHO report highlights the global magnitude of an ever-worsening crisis, exacerbated by a chronic shortage of antibiotics capable of treating infections cause by MDR pathogens^3^. Currently, the majority of drug candidates in the antibiotic pipeline are derivatives of known antibiotics that will provide only a short-term solution to the problem^3^. Furthermore, many of these drug candidates have limited or no activity against MDR Gram-negative bacterial pathogens, which are particularly problematic to treat^3^.

Addressing the global antibiotic resistance problem requires the development of new drug chemotypes and the identification of new antibacterial drug targets. The filamentous temperature-sensitive Z (FtsZ) protein has been identified as a promising new target for the development of novel antibiotics^4–14^. FtsZ has several properties that make it an appealing antibacterial drug target. It is an essential protein required for bacterial division^15–17^. Inhibition of FtsZ has a bactericidal rather than bacteriostatic effect^8,11^, a property that reduces the potential for emergence of future resistance^18^. FtsZ also has no functional human homolog, offering the potential to target this protein specifically and with minimal toxicity^12,19^. It is one of the most abundant and highly conserved cytoskeleton proteins among eubacteria^20^, offering FtsZ inhibitors the potential for broad-spectrum antibacterial activity. Lastly, FtsZ is a “druggable” target whose function can be disrupted by small molecule targeting of a single site on the protein. While this latter property is appealing for a target protein, it also introduces the potential for the development of resistance via single mutations^7,8,11–14,21,22^.

We have previously developed prodrugs of benzamide FtsZ inhibitors (**PC190723** and **TXA707**) that are highly efficacious against infections caused by methicillin-resistant *Staphylococcus aureus* (MRSA)^9–11^. One of these prodrugs (**TXA709**) is currently in phase I clinical trials^6^. To date, the bulk of the compounds that have been validated as FtsZ inhibitors both *in vitro* with purified FtsZ and in bacterial cells are associated with potent activity against staphylococci, *Mycobacterium tuberculosis*, and select other Gram-positive bacterial strains, but weaker or no activity against Gram-negative species^8,11–13,23,24^. Demonstration of *in vivo* efficacy among these FtsZ inhibitors has been limited almost exclusively to the treatment of *S. aureus* and *M. tuberculosis* infections^7–12,14,24,25^.

Advancing the development of new FtsZ inhibitors that can target a more expansive array of both Gram-positive and Gram-negative bacterial pathogens requires tools that allow us to screen for FtsZ inhibition in a broad range of bacterial species. Fluorescent antibiotics are useful tools for delineating the mechanisms underlying the antibacterial activities of compounds as well as the resistance phenotypes of bacteria^26^. In addition, such tools can be used to screen for new antibiotic candidates with desired mechanisms of action^26^. Early efforts aimed at developing fluorescent FtsZ inhibitors were centered on analogs of the benzamide inhibitor **PC190723**^27^. Several of these fluorescent analogs were shown to bind FtsZ from both *S. aureus* and *Bacillus subtilis* (SaFtsZ and BsFtsZ, respectively), though the interactions were weak (with estimated K_d_ values in the range of 11 to 29 µM for BsFtsZ at 25 °C), and none of the analogs were able to bind FtsZ from *Escherichia coli* (EcFtsZ) to a significant degree^27^. One analog was used to visualize FtsZ in *S. aureus* and *B. subtilis* cells^27^. However, visualization required prolonged (1- to 3-hour) treatment with large concentrations of the analog (25 to 200 µM) and was lost upon pre-treatment with the parent inhibitor **PC190723**, limiting the usefulness of the analog as a screening tool for FtsZ inhibitors.

**Figure 1:**
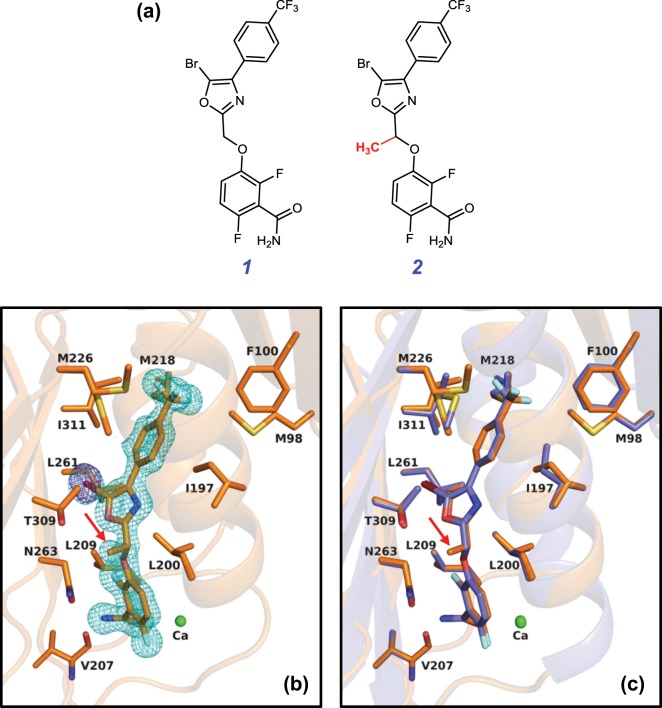
(**a**) Chemical structures of the oxazole-benzamide FtsZ inhibitors **1** and **2**. The methyl group in **2** that differentiates this compound from **1** is highlighted in red. **2** was prepared as a racemic mixture of R and S enantiomers. (**b**) Expanded view of the binding site for the R enantiomer of **2** [**(R)-2**] in complex with SaFtsZ, with the F_0_ – F_c_ omit map (cyan) being contoured at 3.0σ. The anomalous difference map (purple) is contoured at 4.0σ. (**c**) Superposition of the SaFtsZ−**(R)-2** complex (orange) with the corresponding SaFtsZ−**1** complex (blue). The methyl group in **2** shown in red in (**a**) is highlighted by the red arrows in (**b**,**c**).

Here we report the structure-guided design and characterization of a next-generation fluorescent FtsZ probe (**BOFP**) that overcomes the limitations associated with the early-generation analogs. Our design incorporates an oxazole-benzamide FtsZ inhibitor (**1**)^13^ (shown in Fig. 1a), whose crystal structure in complex with SaFtsZ we have previously determined (PDB entry: 5XDU)^21^, conjugated to a boron-dipyrromethene (BODIPY) fluorophore at the linker joining the oxazole and benzamide rings. Fluorescence anisotropy studies demonstrate that **BOFP** can target the FtsZ proteins from a broad range of Gram-positive pathogens (including *S. aureus*, *Enterococcus faecalis*, *Enterococcus faecium*, *Streptococcus pyogenes*, *Streptococcus agalactiae*, and *Streptococcus pneumoniae*) with high affinity (K_d_ values in the range of 1.0 to 3.5 µM at 25 °C). Significantly, **BOFP** targets the FtsZ proteins from clinically important Gram-negative pathogens (including *E. coli*, *Klebsiella pneumoniae*, *Pseudomonas aeruginosa*, and *Acinetobacter baumannii*) with an even higher affinity (K_d_ values in the range of 0.3 to 0.6 µM at 25 °C). The crystal structure of **BOFP** in complex with SaFtsZ confirms that the probe targets the same site on the protein as the parent compound, while revealing contributions from the BODIPY functionality itself to the stability of the complex. Most importantly, fluorescence microscopy studies demonstrate that brief (5-minute) exposure to **BOFP** (at a concentration of only 1.3 µM) can be used to visualize FtsZ in all the Gram-positive and Gram-negative bacterial pathogens listed above, even when pre-treated with other non-fluorescent FtsZ inhibitors. Taken together, our results indicate that **BOFP** can serve as a powerful tool for identifying new broad-spectrum FtsZ inhibitors and understanding their mechanisms of action.

## Results and Discussion

### Structure-guided identification of a suitable site on the oxazole-benzamide FtsZ inhibitor for conjugation of a fluorophore

A clue to a potential site on the oxazole-benzamide FtsZ inhibitor **1** for conjugation of a fluorophore came from the crystal structure we determined for the complex of SaFtsZ_12–316_ (the enzymatic domain of SaFtsZ, residues 12–316) with **2**^13^, a methyl analog of **1** whose chemical structure is shown in Fig. 1a. The only difference between the two compounds is the presence of a methyl group (shown in red in Fig. 1a) on the linker connecting the oxazole and difluorobenzamide rings of **2** that is absent in **1**. This difference makes **2** a chiral molecule, while **1** is achiral. A racemic mixture of the R and S enantiomeric forms of **2** was dissolved in DMSO and introduced into crystals of SaFtsZ_12–316_ by soaking. The structure of the R enantiomer of **2** [**(R)-2**] in complex with SaFtsZ_12–316_ was determined at 1.4 Å resolution. An extra electron density was clearly observed in the cleft between the *N*- and *C*-terminus domains, which enabled us to determine the position and orientation of **(R)-2** with an occupancy of 1.0 (Fig. 1b). We have previously reported the structure of **1** in complex with SaFtsZ_12–316_^21^. **(R)-2** binds the same protein cleft as **1** in a similar orientation to that observed in the structure of the **1**-SaFtsZ_12–316_ complex (see the overlay of **1** and **(R)-2** depicted in Fig. 1c). Note that no SaFtsZ complexes were observed with the S enantiomer of **2**, suggesting that SaFtsZ is selective for the R over the S enantiomer.

Inspection of the crystal structure of **(R)-2** in complex with SaFtsZ_12–316_ reveals that the methyl group distinguishing **(R)-2** from **1** does not engage in significant interactions with SaFtsZ, but rather is oriented away from the cleft of the FtsZ molecule (as highlight by the red arrows in Fig. 1b,c). This observation suggested to us that a bulky fluorescent moiety could be conjugated to the same site on the linker in **1** as the methyl group in **2** without disrupting the FtsZ binding interaction to a significant degree.

### Design of a BODIPY-conjugated fluorescent derivative of 1 (BOFP) that binds SaFtsZ with affinity in the K_d_ range of 0.9 to 3.1 µM

**Figure 2:**
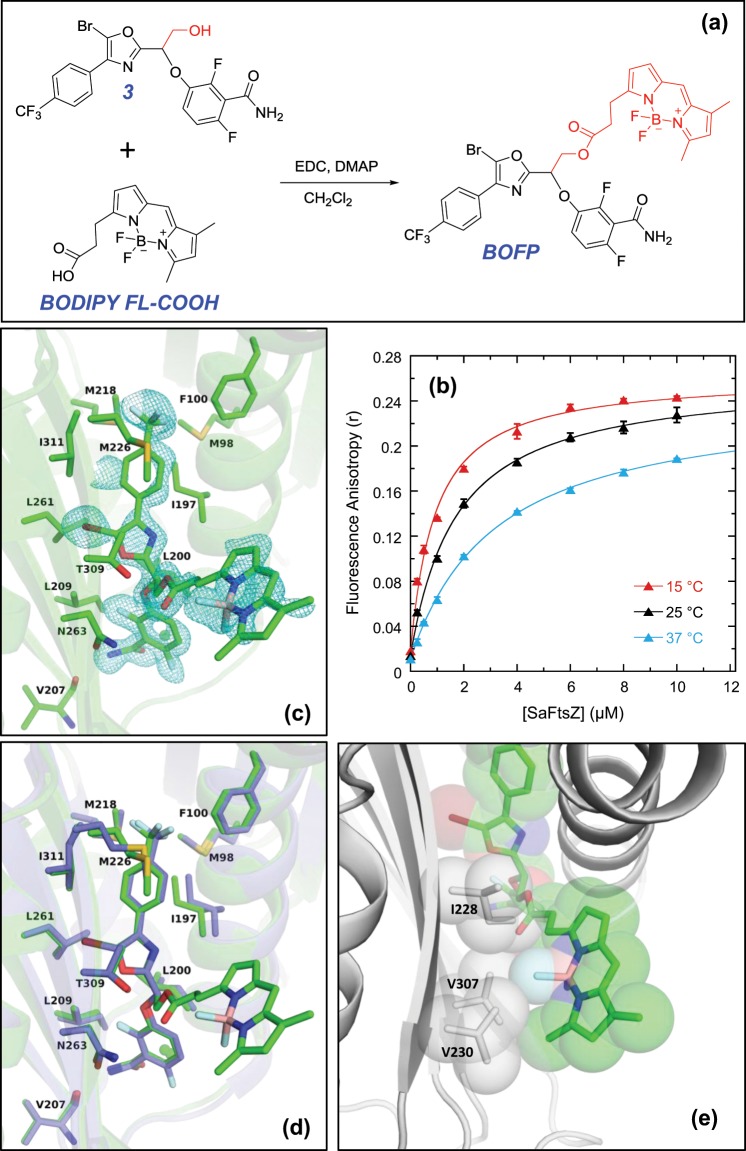
(**a**) Scheme for the synthesis of **BOFP** by reacting **3** with **BODIPY FL-COOH** in CH_2_Cl_2_ containing 1-ethyl-3-(3-dimethylaminopropyl)carbodiimide (EDC) and 4-dimethylaminopyridine (DMAP). Both **3** and **BOFP** were prepared as racemic mixtures of the R and S enantiomers. The tethered hydroxymethyl functionality on **3** and BODIPY functionality on **BOFP** are both highlighted in red. (**b**) Fluorescence anisotropy profiles of 0.1 µM **BOFP** as a function of increasing concentrations of SaFtsZ. The titration experiments were conducted at 15 °C (red), 25 °C (black), or 37 °C (cyan) in solution containing 50 mM Tris-HCl (pH 7.6) and 50 mM KCl. The solid lines reflect non-linear least squares fits of the experimental data points with Eq. 1. (**c**) Expanded view of the binding site for the R enantiomer of **BOFP** [**(R)-BOFP**] in complex with SaFtsZ, with the F_0_ – F_c_ omit map (cyan) being contoured at 2.0σ. (**d**) Superposition of the SaFtsZ−**(R)-BOFP** complex (green) with the corresponding SaFtsZ−**1** complex (blue). (**e**) Hydrophobic interactions at the surface interface between the BODIPY moiety of **BOFP** and residues Ile228, Val230, and Val307 of SaFtsZ.

Armed with the structural results described above, we designed and synthesized **BOFP**, a fluorescent analog of **1** in which a BODIPY fluorophore is conjugated to the desired site on **1** by reaction of BODIPY FL carboxylate (**BODIPY FL-COOH**) with **3**^12,13^ (a hydroxymethyl analog of **1** prepared as a racemic mixture of R and S enantiomers) as schematically depicted in Fig. 2a. This reaction resulted in a racemic mixture of **BOFP** as well. In our initial characterizations, we sought to determine whether **BOFP** could still bind SaFtsZ, as hypothesized in our structure-guided design approach. Toward this end, we used fluorescence anisotropy to monitor the interaction of **BOFP** with SaFtsZ at three different temperatures (15, 25, and 37 °C). Significantly, the fluorescence anisotropy (*r*) of **BOFP** increases markedly with added SaFtsZ (Fig. 2b), indicating the presence of a binding interaction. No such anisotropy change was observed in control studies with unreacted **BODIPY FL-COOH** (Fig. S1). This important control observation not only demonstrates that **BODIPY FL-COOH** does not interact with the target FtsZ protein but also that the ester linkage connecting the BODIPY fluorophore in the **BOFP** conjugate is not hydrolyzed during the binding reactions, as **BODIPY FL-COOH** would be the fluorescent product of such a hydrolysis.

Analysis of the *r*-based binding isotherms of **BOFP** with the 1:1 binding formalism embodied by Eq. 1 yielded excellent fits of the experimental data points (depicted by the solid curves in Fig. 2b) and the corresponding K_d_ values listed in Table 1, which ranged from 0.88 ± 0.08 µM at 15 °C to 3.14 ± 0.13 µM at 37 °C. These gratifying results indicate that **BOFP** can bind SaFtsZ with a robust affinity in the sub- to low-micromolar K_d_ range.

**Table 1:** Equilibrium affinity constants and thermodynamic parameters for the binding of **BOFP** to FtsZ proteins from Gram-positive and Gram-negative bacteria.

FtsZ Protein	^a^K_d_ - 15 °C [µM]	^a^K_d_ - 25 °C [µM]	^a^K_d_ - 37 °C [µM]	^b^ΔH [kcal/mol]	^b^ΔS [cal/mol•*K*]	^c^ΔG [kcal/mol]
**Gram-Positive:**						
*S. aureus* (SaFtsZ)	0.88 ± 0.08	1.66 ± 0.12	3.14 ± 0.13	−10.2 ± 0.3	−7.8 ± 1.0	−7.8 ± 0.1
*E. faecalis* (EfsFtsZ)	1.72 ± 0.06	3.05 ± 0.25	4.62 ± 0.12	−7.9 ± 1.0	−1.2 ± 3.3	−7.6 ± 0.1
*E. faecium* (EfmFtsZ)	2.50 ± 0.14	2.62 ± 0.08	3.14 ± 0.22	−1.9 ± 0.6	+ 19.2 ± 1.9	−7.8 ± 0.1
*S. pyogenes* (SpyFtsZ)	0.91 ± 0.06	1.31 ± 0.08	1.55 ± 0.08	−4.3 ± 1.1	+ 12.8 ± 3.7	−8.2 ± 0.1
*S. agalactiae (*SagFtsZ)	0.62 ± 0.05	1.02 ± 0.09	1.31 ± 0.04	−6.6 ± 0.6	+ 5.4 ± 2.0	−8.2 ± 0.1
*S. pneumoniae* (SpnFtsZ)	3.02 ± 0.30	3.49 ± 0.25	3.81 ± 0.69	−1.9 ± 0.3	+ 18.7 ± 1.2	−7.7 ± 0.1
**Gram-Negative:**						
*E. coli* (EcFtsZ)	0.22 ± 0.03	0.28 ± 0.02	0.44 ± 0.04	−5.6 ± 0.8	+ 11.2 ± 2.7	−9.0 ± 0.1
*K. pneumoniae* (KpFtsZ)	0.42 ± 0.05	0.58 ± 0.04	0.82 ± 0.04	−5.4 ± 0.1	+ 10.5 ± 0.1	−8.6 ± 0.1
*P. aeruginosa* (PaFtsZ)	0.23 ± 0.02	0.36 ± 0.06	0.58 ± 0.06	−7.7 ± 0.2	+ 3.8 ± 0.6	−8.8 ± 0.1
*A. baumannii* (AbFtsZ)	0.40 ± 0.03	0.55 ± 0.02	0.68 ± 0.04	−4.2 ± 0.6	+ 14.5 ± 2.1	−8.8 ± 0.1

^a^K_d_ values (determined at 15, 25, and 37 °C) were derived from non-linear least squares fits of the fluorescence anisotropy profiles shown in Figs. 2, 3, and 5 with Eq. 1, with the indicated uncertainties reflecting the standard deviation of the fitted curves from the experimental data points. ^b^ΔH and ΔS values were derived from linear fits of the ln(1/K_d_) vs. 1/T plots shown in Figs. 3 and 5 with Eq. 3, with the indicated uncertainties reflecting the standard deviation of the fitted lines from the experimental data points. ^c^ΔG values were calculated at T = 310 *K* (37 °C) using Eq. 2 and the corresponding values of K_d_, with the indicated uncertainties reflecting the maximal errors as propagated through that equation.

### The binding of BOFP to SaFtsZ does not require the presence of GTP or magnesium

The filamentation of SaFtsZ requires the presence of both GTP and magnesium^28^. Note that neither of these reagents was present in the fluorescence anisotropy binding studies depicted in Fig. 2b, indicating that the binding of **BOFP** to SaFtsZ does not require the presence of GTP or magnesium. This observation markedly contrasts the fluorescence anisotropy studies previously reported by Artola *et al*.^27^, which demonstrated that the fluorescent analogs of the benzamide FtsZ inhibitor **PC190723** required both GTP and magnesium in order to bind SaFtsZ. For comparative purposes, we sought to determine whether the presence of a non-hydrolyzable analog of GTP (GMPCPP) and magnesium exerted an impact on the binding of **BOFP** to SaFtsZ, as reflected by a change in fluorescence anisotropy. At identical concentrations of **BOFP** (0.1 µM) and SaFtsZ (10 µM), the presence of neither GMPCPP alone (at 0.1 mM) nor MgCl_2_ alone (at 10 mM) has a significant effect on the anisotropy of SaFtsZ-bound **BOFP** (Fig. S2), confirming that the binding of **BOFP** to SaFtsZ is independent of either GTP or magnesium. The presence of both GMPCPP and MgCl_2_ results in a modest increase in the anisotropy of bound **BOFP** (Fig. S2), which likely reflects the filamentation of **BOFP**-bound SaFtsZ induced by the combination of GMPCPP and magnesium.

### BOFP binds in the same cleft of SaFtsZ as 1 and 2

We next sought to determine the crystal structure of **BOFP** in complex with SaFtsZ_12–316_. Toward this end, a racemic mixture of the R and S enantiomeric forms of **BOFP** in DMSO was introduced into crystals of SaFtsZ_12–316_ by soaking. The structure of the R enantiomer of **BOFP** [**(R)-BOFP**] in complex with SaFtsZ_12–316_ was determined at 1.6 Å resolution. Although the electron density of **(R)-BOFP** was not quite as robust as that of **(R)-2** due to a lower occupancy of 0.6, we were able to confirm that **(R)-BOFP** does indeed bind in the same cleft (compare Figs. 1b and 2c). The conjugated BODIPY extends toward the outside of the FtsZ molecule, away from the binding cleft. As hypothesized, interactions between the **1** portion of **(R)-BOFP** and SaFtsZ are similar to those exhibited by **1** (see the overlay of **1** and **(R)-BOFP** depicted in Fig. 2d). In addition to these interactions, the complex of **(R)-BOFP** with SaFtsZ is further stabilized by hydrophobic interactions between the BODIPY moiety and the hydrophobic surface formed by residues Ile228, Val230, and Val307 (Fig. 2e), with these interactions being unlikely to hamper the ability of SaFtsZ to self-associate into filaments (Fig. S3).

As seen with **2**, no SaFtsZ complexes were observed with the S enantiomer of **BOFP**, further suggestive of the selectivity of SaFtsZ for the R versus the S enantiomeric form. Previous studies by Stokes *et al*. have shown that the R enantiomeric form of **3** has significantly greater activity against methicillin-sensitive *S. aureus* (MSSA) than the S enantiomeric form^13^. This enhanced antistaphylococcal activity of the R enantiomer likely reflects the corresponding selectivity of SaFtsZ for the R enantiomeric form.

Note that the FtsZ targeting of **BOFP** confers the compound with antistaphylococcal activity, though this activity is somewhat reduced relative to the parent compounds **1** and **3** (MIC versus MRSA NRS705 = 0.25, 0.5, and 1.0 µg/mL for **1**, **3**, and **BOFP**, respectively). The reduced activities of both **3** and **BOFP** relative to **1** may be due in part to **3** and **BOFP** being racemic mixtures of active R and weakly active S enantiomers. In the aggregate, our collective fluorescence anisotropy, crystallographic, and antibacterial results for **BOFP** serve to validate our structure-guided design approach.

### BOFP can target the FtsZ proteins from a broad range of clinically important Gram-positive bacterial pathogens, including enterococcal and streptococcal species

**Figure 3:**
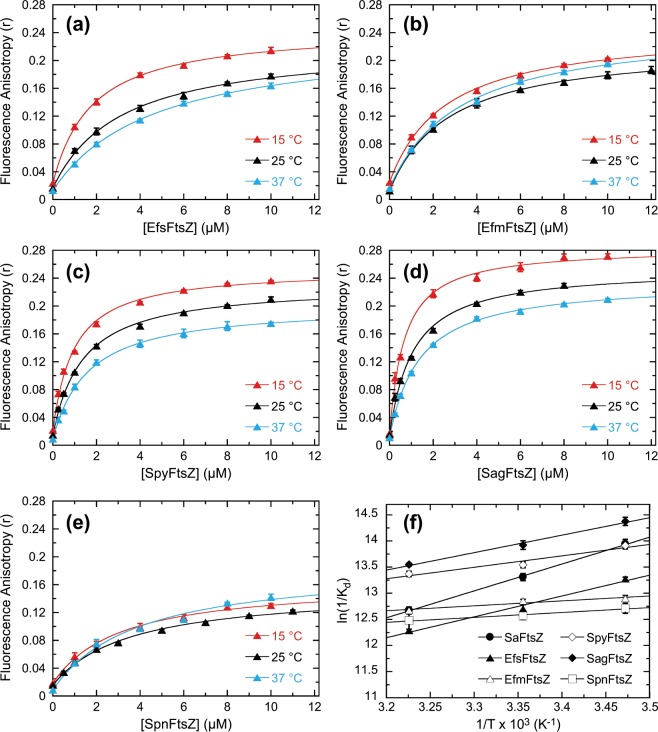
Fluorescence anisotropy profiles of 0.1 µM **BOFP** as a function of increasing concentrations of EfsFtsZ (**a**), EfmFtsZ **(b**), SpyFtsZ (**c**), SagFtsZ (**d**), or SpnFtsZ (**e**). Acquisition and display parameters are as described in the legend to Fig. 2b. Panel (f) shows plots of ln(1/K_d_) vs. 1/T for the interaction of **BOFP** with SaFtsZ (filled circles), EfsFtsZ (filled triangles), EfmFtsZ (open triangles), SpyFtsZ (open diamonds), SagFtsZ (filled diamonds), and SpnFtsZ (open squares). The solid lines reflect linear fits of the experimental data points with Eq. 3.

In addition to SaFtsZ, we also sought to determine whether **BOFP** can target the FtsZ proteins from other Gram-positive bacterial pathogens, including *E. faecalis* (EfsFtsZ) *E. faecium* (EfmFtsZ), *S. pyogenes* (SpyFtsZ), *S. agalactiae* (SagFtsZ), and *S. pneumoniae* (SpnFtsZ). Fluorescence anisotropy studies conducted at 15, 25, and 37 °C reveal that addition of each of the five target FtsZ proteins increases the anisotropy of **BOFP** significantly (Fig. 3a–e), indicative of a binding interaction between the probe and each of the host proteins. Significantly, no such binding interactions were observable with **BODIPY FL-COOH** (as exemplified by the SpyFtsZ results shown in Fig. S1). Thus, **BOFP** can target not only SaFtsZ, but also the FtsZ proteins from a broad range of other clinically important Gram-positive pathogens.

Analysis of the anisotropy isotherms in Fig. 3 with Eq. 1 yielded outstanding fits of the experimental data points (as depicted by the solid curves), with the K_d_ values derived from these fits being listed in Table 1. K_d_ values for **BOFP** binding to the Gram-positive FtsZ proteins ranged from 1.72 ± 0.06 µM at 15 °C to 4.62 ± 0.12 µM at 37 °C for EfsFtsZ, 2.50 ± 0.14 µM at 15 °C to 3.14 ± 0.22 µM at 37 °C for EfmFtsZ, 0.91 ± 0.06 µM at 15 °C to 1.55 ± 0.08 µM at 37 °C for SpyFtsZ, 0.62 ± 0.05 µM at 15 °C to 1.31 ± 0.04 µM at 37 °C for SagFtsZ, and 3.02 ± 0.30 µM at 15 °C to 3.81 ± 0.69 µM at 37 °C for SpnFtsZ. These K_d_ ranges are similar in magnitude to that observed for SaFtsZ. Recall that Artola *et al*. demonstrated the binding of their early-generation fluorescent FtsZ inhibitors only to SaFtsZ and BsFtsZ, while reporting K_d_ values in the range of 11 to 29 µM for BsFtsZ at 25 °C^27^. Significantly, the lower range of K_d_ values we observe for the binding of **BOFP** to all six Gram-positive FtsZ proteins at 25 °C (1.02 ± 0.09 to 3.49 ± 0.25 µM) indicates a much broader spectrum of FtsZ targeting as well as a binding affinity that is approximately 3- to 29-fold higher.

### Brief exposure to a low concentration of BOFP effectively labels FtsZ in live *S. aureus*, *E. faecalis*, *E. faecium*, *S. pyogenes*, *S. agalactiae*, and *S. pneumoniae* cells

**Figure 4:**
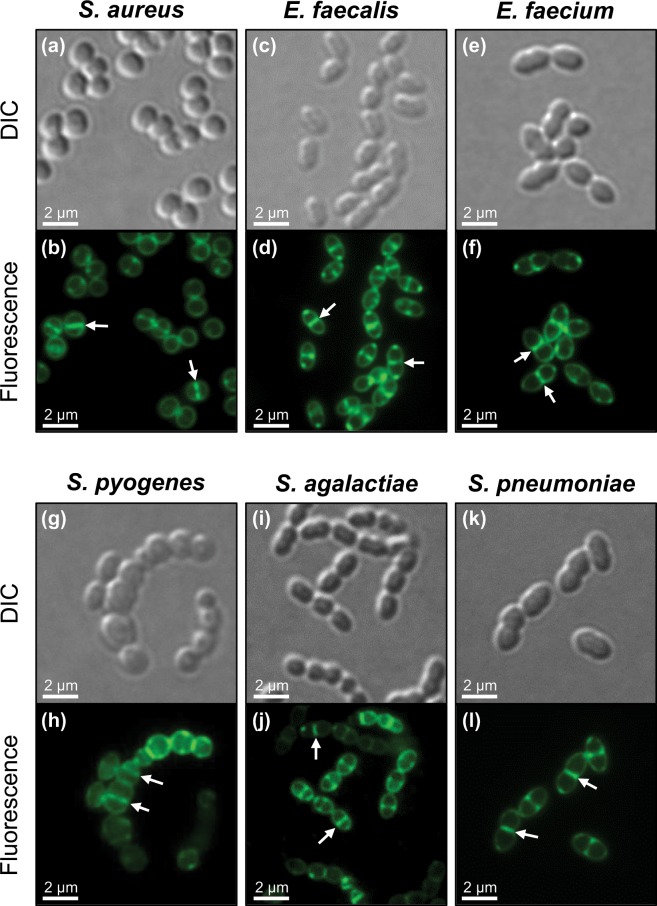
FtsZ visualization in the Gram-positive bacterial pathogens *S. aureus* NRS705 (**a**,**b**), *E. faecalis* ATCC 29212 (**c**,**d**), *E. faecium* ATCC 19434 (**e**,**f**), *S. pyogenes* ATCC 19615 (**g**,**h**), *S. agalactiae* ATCC 12386 (**i**,**j**), and *S. pneumoniae* ATCC 49619 (**k**,**l**). Differential interference contrast (DIC) and fluorescence micrographs of the indicated bacterial cells treated for 5 minutes with 1 µg/mL **BOFP** just prior to visualization. The arrows in panels (b,d,f,h,j,l) highlight representative FtsZ Z-rings at midcell labeled by **BOFP**.

Having demonstrated the high-affinity binding of **BOFP** to the Gram-positive FtsZ proteins SaFtsZ, EfsFtsZ, EfmFtsZ, SpyFtsZ, SagFtsZ, and SpnFtsZ, we explored the potential of the probe to label FtsZ in live cells of the corresponding pathogens themselves. Figure 4 shows differential interference contrast (DIC) and fluorescence micrographs of *S. aureus*, *E. faecalis*, *E. faecium*, *S. pyogenes*, *S. agalactiae*, and *S. pneumoniae* cells labeled for 5 minutes with 1 µg/mL (1.3 µM) **BOFP**. In each of the six pathogens, bright bands of fluorescence staining are clearly visible at midcell (as highlighted by the arrows in Fig. 4b,d,f,h,j,l), consistent with the labeling of FtsZ Z-rings. Additional fluorescence staining (though weaker than that at midcell) is also evident along the periphery of each cell, suggesting that FtsZ is also localized throughout the cell membrane. These results indicate that brief exposure to a low concentration of **BOFP** affords outstanding visualization of FtsZ and its localization patterns in live Gram-positive bacterial cells.

To ensure that the ester linkage connecting the BODIPY fluorophore in the **BOFP** conjugate was not being hydrolyzed by the bacterial cells, we examined the potential, if any, of **BODIPY FL-COOH** to label FtsZ in *S. aureus* cells. Significantly, no fluorescence staining of the *S. aureus* cells was detectable upon treatment with 1 µg/mL **BODIPY FL-COOH** for 5 minutes (Fig. S4). This behavior markedly contrasts the clear staining patterns observed when the cells are treated similarly with **BOFP** (Fig. S4). These results confirm that **BOFP** is not hydrolyzed by the cells during the treatment regimen and that the staining patterns observed were not the result of nonspecific interactions with a fluorescent product of hydrolysis.

### BOFP targets FtsZ proteins from Gram-negative bacterial pathogens with an even higher affinity than FtsZ proteins from Gram-positive pathogens

**Figure 5:**
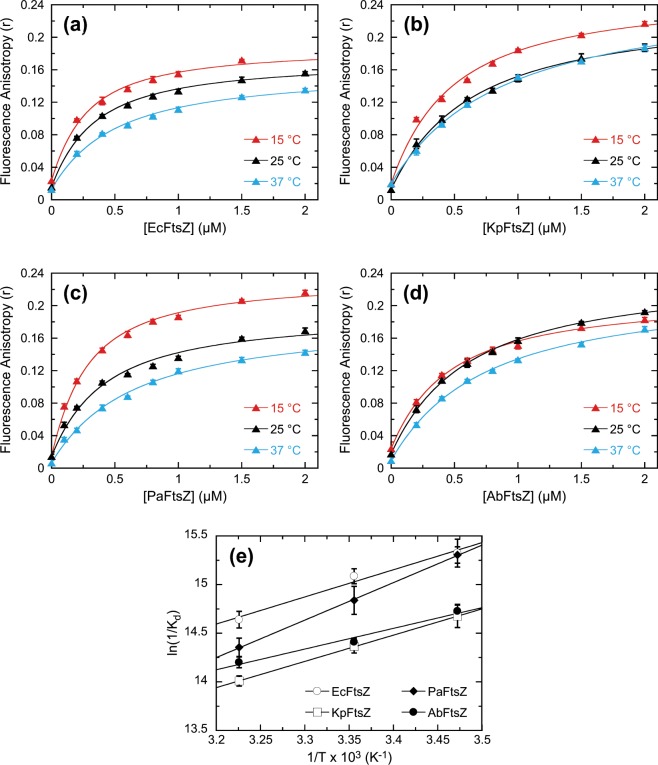
Fluorescence anisotropy profiles of 0.1 µM **BOFP** as a function of increasing concentrations of EcFtsZ (**a**), KpFtsZ (**b**), PaFtsZ (**c**), or AbFtsZ (**d**). Acquisition and display parameters are as described in the legend to Fig. 2b. Panel (e) shows plots of ln(1/K_d_) vs. 1/T for the interaction of **BOFP** with EcFtsZ (open circles), KpFtsZ (open squares), PaFtsZ (filled diamonds), and AbFtsZ (filled circles). The solid lines reflect linear fits of the experimental data points with Eq. 3.

In addition to targeting Gram-positive FtsZ proteins, we sought to determine whether **BOFP** could also target Gram-negative FtsZ proteins. To this end, we used fluorescence anisotropy to explore the interactions of **BOFP** with the FtsZ proteins from the four Gram-negative pathogens, *E. coli* (EcFtsZ), *K. pneumoniae* (KpFtsZ), *P. aeruginosa* (PaFtsZ), and *A. baumannii* (AbFtsZ), with the resulting anisotropy profiles acquired at 15, 25, and 37 °C being depicted in Fig. 5a–d. Inspection and analysis of these anisotropy profiles reveals that **BOFP** binds to all four Gram-negative FtsZ proteins with sub-micromolar affinity. At 25 °C, the K_d_ values for EcFtsZ, KpFtsZ, PaFtsZ, and AbFtsZ are 0.28 ± 0.02, 0.58 ± 0.04, 0.36 ± 0.06, and 0.55 ± 0.02 µM, respectively (Table 1). A comparison of the K_d_ values at 25 °C listed in Table 1 indicates that **BOFP** binds the Gram-negative FtsZ proteins with an approximately 2- to 12-fold higher affinity than the Gram-positive FtsZ proteins. Thus, in striking contrast to the early-generation fluorescent FtsZ inhibitors reported by Artola *et al*.^27^, **BOFP** can target a broad range of Gram-negative FtsZ proteins with a high degree of affinity. As observed with the Gram-positive FtsZ proteins, no binding interactions with **BODIPY FL-COOH** were detectable with the Gram-negative FtsZ proteins (as exemplified by the KpFtsZ and PaFtsZ results shown in Fig. S1).

### For the majority of target FtsZ proteins studied, enthalpy provides a significant driving force for the binding of BOFP

We used the temperature dependence of the K_d_ values for the binding of **BOFP** to the Gram-positive and Gram-negative FtsZ proteins to derive the thermodynamic parameters associated with the binding reactions. Free energy changes (ΔG) at 37 °C (310 K) were derived from the corresponding K_d_ values using Eq. 2, while enthalpy and entropy changes (ΔH and ΔS, respectively) were determined from linear fits of the ln(1/K_d_) vs. 1/T plots shown in Figs. 3f and 5e with Eq. 3. The resulting thermodynamic parameters are listed in Table 1. For seven of the ten FtsZ proteins studied (SaFtsZ, EfsFtsZ, SpyFtsZ, SagFtsZ, EcFtsZ, KpFtsZ, and PaFtsZ), ΔH contributes >50% to the observed ΔG of binding, with the enthalpic contribution to binding being 100% for two of those seven FtsZ proteins (SaFtsZ and EfsFtsZ). As suggested by our crystal structure of **BOFP** in complex with SaFtsZ, these favorable enthalpic contributions to binding likely stem from the extensive array of favorable van der Waals contacts between the host protein and both the **1** and BODIPY portions of the probe (Fig. 2c–e). For the remaining three FtsZ proteins (EfmFtsZ, SpnFtsZ, and AbFtsZ), ΔS contributes >50% to the observed ΔG of binding. These favorable entropic contributions to binding may reflect favorable binding-induced changes in hydration and/or conformation of the host proteins.

### In addition to Gram-positive bacterial cells, **BOFP** also labels FtsZ effectively in live Gram-negative cells of *E. coli*, *K. pneumoniae*, *P. aeruginosa*, and *A. baumannii*

**Figure 6:**
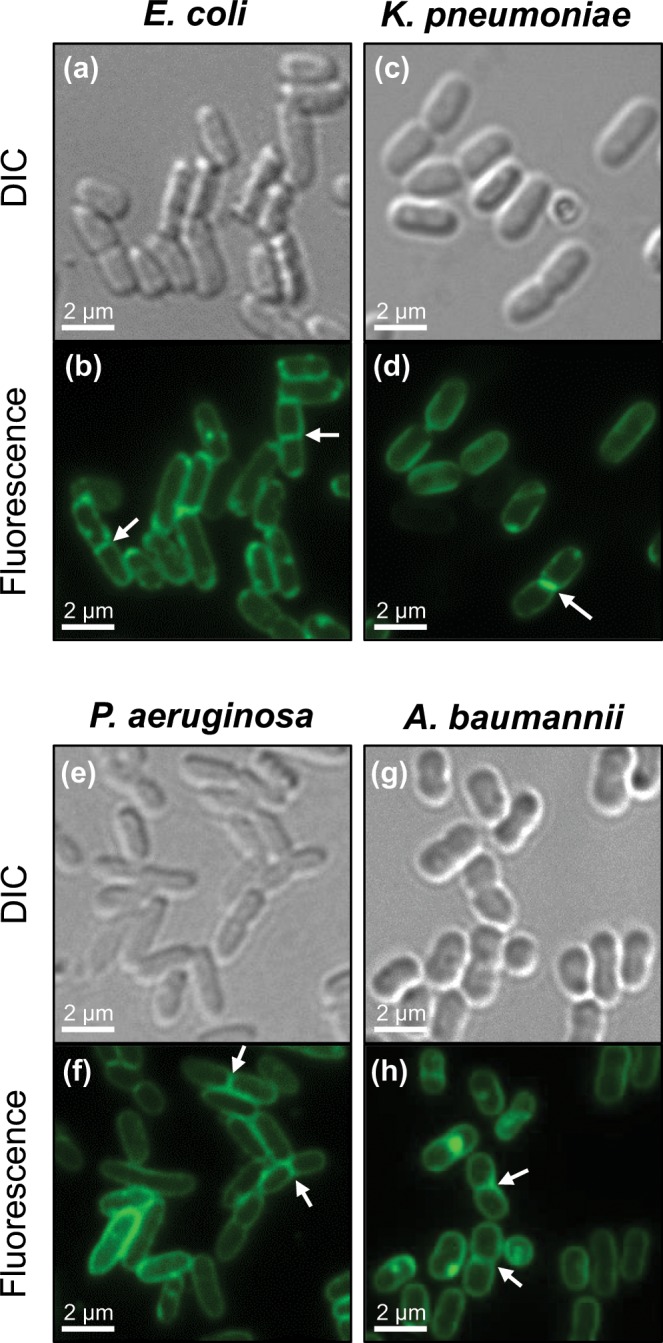
FtsZ visualization in the Gram-negative bacterial pathogens *E. coli* ATCC 25922 (**a**,**b**), *K. pneumoniae* ATCC 13883 (**c**,**d**), *P. aeruginosa* ATCC 27853 (**e**,**f**), and *A. baumannii* ATCC 19606 (**g**,**h**). Differential interference contrast (DIC) and fluorescence micrographs of the indicated bacterial cells treated for 5 minutes with 1 µg/mL **BOFP** in the presence of pentamidine isethionate (at 0.875 mg/mL for *E. coli* and 3.5 mg/mL for the other three strains) just prior to visualization. The arrows in panels (b,d,f,h) highlight representative FtsZ Z-rings at midcell labeled by **BOFP**.

Armed with knowledge that **BOFP** binds to the Gram-negative FtsZ proteins EcFtsZ, KpFtsZ, PaFtsZ, and AbFtsZ with an even higher affinity than any of the Gram-positive FtsZ proteins, we further explored the potential of the probe to label FtsZ in live Gram-negative bacterial cells. When exposed to **BOFP** in a similar manner (1 µg/mL for 5 minutes) to that described above for the Gram-positive bacteria, little or no FtsZ labeling in the Gram-negative cells was observable by fluorescence microscopy (as exemplified by the *K. pneumoniae* results shown in Fig. S5b). Previous studies have indicated that the large size of fluorescent antibiotics resulting from the conjugation of bulky fluorophores restricts the passage of the agents across the outer membrane of Gram-negative cells^29^. Stokes *et al*. have shown that pentamidine can effectively permeabilize the outer membrane of Gram-negative bacterial cells to large antibiotics that would normally be unable to cross the membrane^30^. When co-treating *K. pneumoniae* cells with 1 µg/mL **BOFP** and 3.5 mg/mL pentamidine isethionate for 5 minutes, bright fluorescence staining becomes visible both at midcell and along the cell periphery (compare Fig. S5b,d). Co-treatment of *E. coli*, *P. aeruginosa*, and *A. baumannii* cells results in a similar fluorescence staining pattern to that observed in *K. pneumoniae* cells (Fig. 6b,d,f,h). The fluorescent bands visible at midcell are consistent with the labeling of FtsZ Z-rings (highlighted by the arrows in Figs. S5d and 6b,d,f,h), with the peripheral staining reflecting the presence of FtsZ in the cell membrane as well. Note that no such FtsZ staining was detectable in *K. pneumoniae* cells co-treated for 5 minutes with 1 µg/mL **BODIPY FL-COOH** and 3.5 mg/mL pentamidine isethionate (Fig. S6), thus confirming that **BOFP** is not being hydrolyzed by the Gram-negative cells during the labeling procedure. Viewed as a whole, our results indicate that **BOFP** is useful for visualizing FtsZ not only in live Gram-positive cells, but also in live Gram-negative cells.

### BOFP can also be used to visualize the impact of non-fluorescent FtsZ inhibitors on the localization of FtsZ in both Gram-positive and Gram-negative bacterial cells

**Figure 7:**
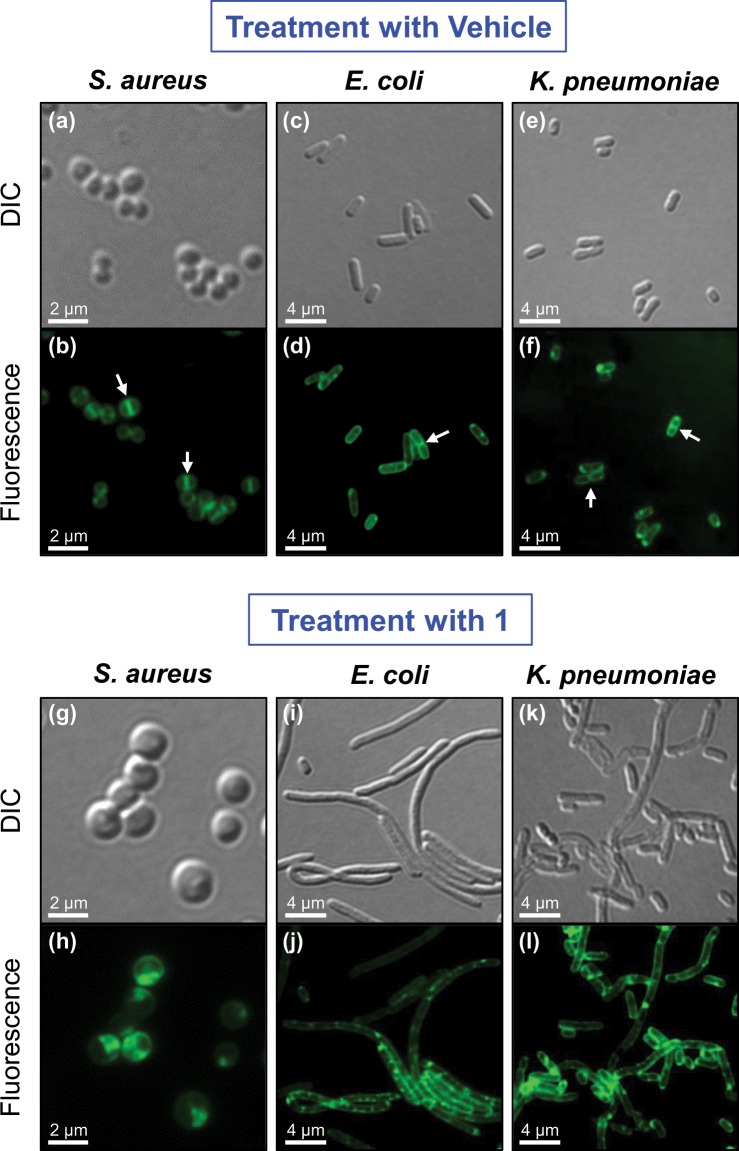
Visualization of the impact of treatment with **1** on FtsZ localization in *S. aureus* NRS705, *E. coli* N43, and *K. pneumoniae* ATCC 10031. Differential interference contrast (DIC) and fluorescence micrographs of the indicated bacterial cells treated for 3 hours with either DMSO vehicle (**a**–**f**) or **1** (**g**–**l**) at 4× MIC (1 µg/mL for *S. aureus* and 4 µg/mL for *E. coli* and *K. pneumoniae*). Just prior to visualization, cells were labeled for 5 minutes with 1 µg/mL **BOFP** in the absence (for *S. aureus*) or presence of pentamidine isethionate (at 0.875 mg/mL for *E. coli* and 3.5 mg/mL for *K. pneumoniae*). The arrows in panels (b,d,f) highlight representative FtsZ Z-rings at midcell labeled by **BOFP**.

For **BOFP** to be useful as a tool for identifying new FtsZ inhibitors in a live cell-based assay, it must facilitate the detection of changes in FtsZ localization induced by non-fluorescent test compounds with the potential for FtsZ inhibition. Toward this end, we tested the ability of **BOFP** to visualize the impact of the oxazole-benzamide FtsZ inhibitor **1** on FtsZ localization in live *S. aureus*, *E. coli*, and *K. pneumoniae* cells, with the results being shown in Fig. 7. As expected with a known FtsZ inhibitor, treatment with **1** (at 4x MIC for 3 hours) induces a significant change in cell morphology consistent with the impairment of cell division (compare Fig. 7a,c,e with Fig. 7g,i,k). This morphological change takes the form of cell enlargement in cocci like *S. aureus* and filamentation in rods like *E. coli* and *K. pneumoniae*. Significantly, **BOFP** effectively labels FtsZ in the cells treated with **1**, showing clear mislocalization of FtsZ and an absence of Z-rings in any of the treated cells (Fig. 7h,j,l). In addition, the presence of FtsZ in the membranes of cells treated with **1** is reduced relative to that in the membranes of untreated cells. In *S. aureus*, we observed similar results for cells treated with the benzamide FtsZ inhibitors **PC190723** and **TXA707** (Fig. S7).

**Figure 8:**
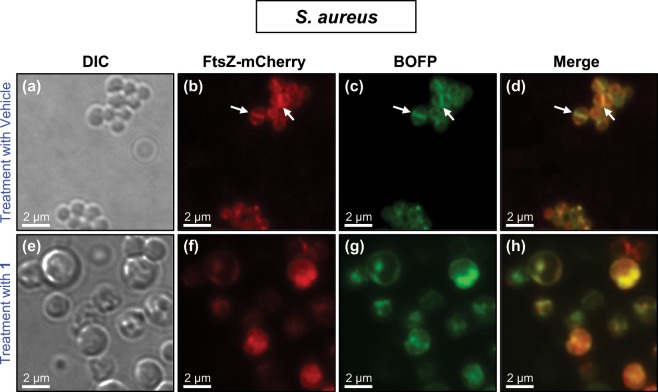
Comparison of **BOFP** and induced expression of the FtsZ-mCherry fusion protein for visualization of the impact of treatment with **1** on FtsZ localization in the MRSA LAC FtsZ-mCherry strain. Differential interference contrast (DIC) and fluorescence micrographs of the bacterial cells treated for 3 hours with either DMSO vehicle (**a**–**d**) or **1** (**e**–**h**) at 4× MIC (0.25 µg/mL). Just prior to visualization, cells were labeled for 5 minutes with 1 µg/mL **BOFP**. The arrows in panel panels (b,c,d) highlight representative FtsZ Z-rings at midcell as visualized either by induced expression of FtsZ-mCherry or by labeling with **BOFP**.

We sought to verify that the **BOFP** staining observed in *S. aureus* cells treated with non-fluorescent FtsZ inhibitors still reflected specific interaction with FtsZ and not off-target interactions. To this end, we used **BOFP** to monitor the impact of treatment with **1** on FtsZ localization in live MRSA LAC cells that expresses a FtsZ-mCherry fusion protein (MRSA LAC FtsZ-mCherry). As shown in Fig. 8, the green fluorescence associated with **BOFP** labeling colocalizes with the red fluorescence associated with the induced expression of FtsZ-mCherry in both vehicle-treated and **1**-treated cells. Thus, the observed BOFP labeling reflects specific interactions with FtsZ even in cells treated with non-fluorescent FtsZ inhibitors.

Our collective results highlight the usefulness of **BOFP** as a screening tool for identifying novel FtsZ inhibitors in both Gram-positive and Gram-negative pathogens. The fluorescent FtsZ inhibitors previously reported by Artola *et al*. lacked this utility, as their ability to label FtsZ was significantly diminished in live cells treated with known FtsZ inhibitors like **PC190723**^27^.

## Conclusions

This work describes the structure-guided design of a fluorescent FtsZ-targeting probe (**BOFP**) consisting of an oxazole-benzamide FtsZ inhibitor conjugated to a BODIPY fluorophore. Crystallographic studies demonstrate that **BOFP** targets the same FtsZ site as inhibitors like **PC190723** and **TXA707**, while also highlighting favorable contributions from the conjugated BODIPY moiety itself to the FtsZ binding reaction. Fluorescence anisotropy studies with **BOFP** provide the first demonstration of a fluorescent probe capable of targeting the FtsZ proteins from a broad range of clinically relevant Gram-positive and Gram-negative bacterial pathogens with a high degree of affinity. In addition, fluorescence microscopy studies highlight the utility of **BOFP** for visualizing FtsZ in these pathogenic bacteria as well as for visualizing the impact of non-fluorescent inhibitors on FtsZ localization. These properties make **BOFP** a robust tool for identifying new broad-spectrum FtsZ inhibitors and understanding their mechanisms of action.

## Materials and Methods

### Bacterial strains and other reagents

*S. aureus* NRS705 (a USA100 MRSA strain isolated from the blood of a 14-day-old male in New York) was provided by the Network on Antimicrobial Resistance in *Staphylococcus aureus* (NARSA) for distribution by BEI Resources, NIAID, NIH. *E. coli* N43 *acrA*^−^ was a generous gift from Dr. Lynn Zechiedrich (Baylor College of Medicine, Houston, TX). MRSA LAC FtsZ-mCherry was generated as detailed in the Supplementary Information. All other bacterial strains were obtained from the American Type Culture Collection (ATCC). MRS, cation-adjusted Mueller-Hinton (CAMH), and tryptic soy media were from Becton-Dickinson. Todd Hewitt (TH) media was made from components specified by Becton-Dickinson. Lysed horse blood (LHB) was from Hardy Diagnostics, the sodium salt of 2′-deoxyguanosine-5′-[(α,β)-methyleno]triphosphate (GMPCPP) was from Jena Bioscience, and phosphate-buffered saline (PBS) was from Lonza. Pentamidine isethionate, isopropyl β-d-1-thiogalactopyranoside (IPTG), and **PC190723** were from Sigma. 3-(5,5-difluoro-7,9-dimethyl-5*H*-5λ^4^,6λ^4^-dipyrrolo[1,2-*c*:2′,1′-*f*][1,3,2]diazaborinin-3-yl)propanoic acid (**BODIPY FL-COOH**) was from Lumiprobe Corp.

### Compound synthesis

**1**, **2**, **3**, and **TXA707**, were synthesized as previously described^11–13,31^. **BOFP** was synthesized as detailed in the Supplementary Information.

### Crystallization and structure determination of SaFtsZ in complex with 2 and BOFP

SaFtsZ_12–316_ was cloned, expressed, and purified as described previously^21^. After SaFtsZ_12–316_ was crystallized by the sitting-drop vapor-diffusion technique at 20 °C in reservoir conditions based on the JBScreen pentaerythritol 2 (Jena Bioscience), racemic mixtures of the R and S enantiomeric forms of **2** or **BOFP** were introduced by soaking. For the complex of **2** with SaFtsZ, the protein was crystallized at 9.5 mg/mL under conditions of 100 mM Tris-HCl (pH 7.8), 45% (w/v) pentaerythritol propoxylate 629 (PEP629), and 300 mM KCl. After 3 weeks, the crystal was soaked in the same reservoir supplemented with 5 mM **2** for 3 days. For the **BOFP** complex, the protein was crystallized at 20 mg/mL under conditions of 100 mM HEPES (pH 8.0) and 39% (w/v) PEP629. After 7 days, the crystal was soaked in the same reservoir supplemented with 1.4 mM **BOFP** for 3 days.

The crystal of the **2**-SaFtsZ complex was flash-frozen in a nitrogen gas stream at −180 °C without cryoprotectants. The crystal of the **BOFP**-SaFtsZ complex was briefly soaked in a cryoprotectant solution containing 100 mM HEPES (pH 8.0), 39% (w/v) PEP629, and 25% (v/v) glycerol and then flash-frozen in the same manner as described above. X-ray diffraction data from the crystals of the **2**-SaFtsZ and **BOFP**-SaFtsZ complexes were collected at SPring-8 BL44XU (Hyogo, Japan) under a cryogenic nitrogen gas stream at 100 K. Diffraction data were processed and scaled with HKL2000^32^ and XDS^33^, respectively. The phases for both complexes were determined by molecular replacement with Phaser in the CCP4 suite^34^ using the previously determined structure of the SaFtsZ-GDP complex (PDB entry: 3VOA)^35^ as a search model. Both models were refined with REFMAC5^36^ and PHENIX^37^, with manual modification using Coot^38^. The refined structures were validated with MolProbity^39^. Data collection and refinement statistics are summarized in Table 2. The final atomic coordinates and structure factor amplitudes have been deposited in the RCSB Protein Data Bank (PDB entries: 6KVP and 6KVQ). Figures were prepared with PyMOL (Schrödinger).

**Table 2:** Data collection and refinement statistics for the crystal structures of SaFtsZ_12–316_ in complex with the R enantiomers of **2** and **BOFP**.

Data Set	^a^SaFtsZ Complex with 2	^a^SaFtsZ Complex with BOFP
PDB entry	6KVP	6KVQ
***Data Collection***		
X-ray source	SPring-8 BL44XU	SPring-8 BL44XU
wavelength	0.900	0.900
space group	*C*2	*C*2
**Unit -Cell Parameters**		
*a, b, c* (Å)	70.49, 51.74, 86.74	72.27, 49.69, 88.59
*β* (deg)	108.65	111.24
resolution (Å)	50.0–1.40 (1.42–1.40)	36.1–1.60 (1.66–1.60)
total reflections	360,570	265,092
unique reflections	58,167	38,791
completeness (%)	99.2 (100.0)	99.8 (100.0)
*I/σ*	26.7 (2.4)	15.9 (2.0)
*R*_merge_ (%)	7.2 (70.4)	6.3 (82.2)
CC_1/2_ (%)	(87.1)	(71.9)
***Refinement***		
resolution (Å)	41.1–1.40	36.1–1.60
*R*_work_/*R*_free_ (%)	14.5/17.7	19.0/21.4
no. of chain in the asymmetric unit	1	1
**No. of Atoms**		
protein	2,387	2,234
ligand	59	80
water	340	165
**Average B-Factors (Å**^2^**)**		
protein	20.6	31.7
ligand	14.6	28.2
water	34.8	37.8
**rmsd from Ideal**		
bond length (Å)	0.007	0.008
bond angle (deg)	1.43	1.28
**Ramachandran Plot (%)**		
favored	97.9	98.4
allowed	2.1	1.6
outlier	0	0

^a^Values in parentheses are for the highest resolution shells.

### Fluorescence anisotropy assays for the binding of BOFP to FtsZ proteins

Fluorescence anisotropy experiments were performed using an AVIV model ATF105 spectrofluorometer at 15, 25, or 37 °C. In these experiments, bandwidths were set to 4 nm in both the excitation and emission directions, with the excitation and emission wavelengths being set at 488 nm and 510 nm, respectively. **BOFP** (0.1 µM) was titrated with increasing concentrations (ranging from 0 to 12 µM) of FtsZ in 120 µL of buffer containing 50 mM Tris-HCl (pH 7.6) and 50 mM KCl. After each protein addition, the samples were equilibrated for 3 minutes, whereupon the fluorescence anisotropy was measured.

Plots of the fluorescence anisotropy (*r*) of **BOFP** as a function of FtsZ concentration (as shown in Figs. 2, 3, and 5) were analyzed by non-linear least squares regression using the following 1:1 binding formalism:

1 $$r={r}_{0}+\frac{{r}_{\infty }-{r}_{0}}{2{[C]}_{tot}}[({[C]}_{tot}+{[P]}_{tot}+{K}_{d})-\sqrt{{({[C]}_{tot}+{[P]}_{tot}+{K}_{d})}^{2}-4{[C]}_{tot}{[P]}_{tot}}]$$

In this equation, *r*_0_ is the anisotropy of the protein-free compound, *r*_∞_ is the anisotropy of the compound in the presence of an infinite concentration of FtsZ, [C]_tot_ is the total concentration of the compound, and [P]_tot_ is the total concentration of protein with each addition. These analyses yielded the equilibrium dissociation constant (K_d_) for each binding reaction.

The binding free energy (ΔG) at temperature T was derived from the corresponding K_d_ value determined at T using the following relationship:

2 $$\Delta G=-\,RTln(\frac{1}{{K}_{d}})$$

The binding enthalpy (ΔH) and entropy (ΔS) were derived from linear fits of the ln(1/K_d_) vs. 1/T plots shown in Figs. 3f and 5e using the following relationship:

3 $$\mathrm{ln}(\frac{1}{{K}_{d}})=-\,\frac{\Delta H}{R}(\frac{1}{T})+\frac{\Delta S}{R}$$

The impact of guanosine nucleotide and magnesium on the binding of **BOFP** to SaFtsZ was assessed at 37 °C in the same buffer described above. In these studies, the anisotropy of 0.1 µM **BOFP** alone or in the presence of 10 µM SaFtsZ was measured, with the latter also being measured in the presence of 0.1 mM GMPCPP (a non-hydrolyzable GTP analog), 10 mM MgCl_2_, or both.

In comparative control experiments, the anisotropy of 0.1 µM **BODIPY FL-COOH** or **BOFP** alone or in the presence of SaFtsZ (10 µM), SpyFtsZ (10 µM), KpFtsZ (2 µM), or PaFtsZ (2 µM) was measured at 37 °C in the same buffer described above.

A quartz ultra-micro cell (Hellma) with a 2 × 5 mm aperture and a 15 mm center height was used for all measurements. The pathlengths in the excitation and emissions directions were 1 and 0.2 cm, respectively. All steady-state anisotropy experiments were conducted in at least triplicate, with the reported anisotropies reflecting the average values.

### Minimum inhibitory concentration (MIC) Assays

MIC assays of **1**, **3**, and **BOFP** were conducted by standard broth microdilution in TH media. Briefly, log-phase *S. aureus* NRS705 (MRSA) cells were added to 96-well microtiter plates (at 5 × 10^5^ CFU/mL) containing 2-fold serial dilutions of each test compound in 0.1 mL of TH broth, with each compound concentration being present in duplicate. The MIC is defined as the lowest compound concentration at which growth is ≥90% inhibited after 18-24 hours of aerobic growth.

### Differential interference contrast and fluorescence microscopy

All differential interference contrast (DIC) and fluorescence microscopy experiments were conducted using an Olympus BX50 microscope equipped with an X-cite Exacte 200 W mercury lamp, a 100× Olympus UPLSAPO oil immersion objective (1.40 aperture), as well as both Chroma ET-EGFP (FITC/Cy2) and ET mCherry, Texas Red filters. Images were captured using a QImaging Retiga R3 charge-coupled device (CCD) camera and the Ocular-Version 2.0 software package (QImaging).

For visualizing FtsZ in Gram-positive bacteria using **BOFP**, the bacterial cells were grown to log-phase in media suitable for each individual pathogen. Specifically, *S. aureus* NRS705 (MRSA) was grown in tryptic soy broth (TSB), *E. faecalis* ATCC 29212 and *E. faecium* ATCC 19434 were grown in lactobacilli MRS broth, *S. agalactiae* ATCC 12386 and *S. pneumoniae* ATCC 49619 were grown in TH broth, and *S. pyogenes* ATCC 19615 was grown in CAMH broth supplemented with 3% (v/v) LHB. For each Gram-positive bacterial strain, a total of 1 mL of cell culture was centrifuged at 15,000 × g for 1 minute and washed 2–3 times with 1 mL of PBS. After the final wash, the pelleted cells were resuspended in 500 µL of PBS containing 1 µg/mL of **BOFP** and incubated in the dark for 5 minutes at room temperature. The cells were then centrifuged at 15,000 × g for 1 minute, washed twice with 1 mL of PBS, and subsequently resuspended in 200 µL of PBS. 8 µL of this final cell suspension was then spread on a 0.25 mm layer of 1.5% high-resolution agarose (Sigma) in PBS, which was mounted on a standard 75 × 25 × 1 mm microscope slide (Azer Scientific) using a 1.7 × 2.8 × 0.025 cm Gene Frame (ThermoFisher). A 24 × 40 mm cover slip (Azer Scientific) was then applied to the agarose pad to prepare the slide for microscopic visualization. Comparative control experiments with 1 µg/mL **BODIPY FL-COOH** were conducted in *S. aureus* NRS705 cells as described above for **BOFP**.

For visualizing FtsZ in Gram-negative bacteria using **BOFP**, *E. coli* ATCC 25922, *K. pneumoniae* ATCC 13883, *P. aeruginosa* ATCC 27853, and *A. baumannii* ATCC 19606 were grown to log-phase in CAMH broth. For each Gram-negative bacterial strain, a total of 1 mL of cell culture was centrifuged at 15,000 × g for 1 minute and washed twice with 1 mL of Tris-buffered saline (TBS) composed of 50 mM Tris-HCl (pH 7.6) and 150 mM NaCl. After the final wash, the pelleted cells were resuspended in 500 µL of TBS containing 1 µg/mL of **BOFP** and pentamidine isethionate (at 0.875 mg/mL for *E. coli* and 3.5 mg/mL for the other three strains). The resuspended cells were then incubated in the dark for 5 minutes at room temperature, centrifuged at 15,000 × g for 1 minute, washed twice with 1 mL of TBS, and subsequently resuspended in 200 µL of TBS. This final cell suspension was then prepared for microscopy as described for the Gram-positive bacterial strains. Comparative control experiments with 1 µg/mL **BODIPY FL-COOH** were conducted in *K. pneumoniae* ATCC 13883 cells as described above for **BOFP**.

To visualize the impact of treatment with the FtsZ inhibitor **1** using **BOFP**, *S. aureus* NRS705 (MRSA), *E. coli* N43, and *K. pneumoniae* ATCC 10031 were grown to log-phase in CAMH broth and diluted to an OD_600_ of 0.1. Each cell culture was then treated with either DMSO vehicle or **1** at 4× MIC (1 µg/mL for *S. aureus* or 4 µg/mL for *E. coli* and *K. pneumoniae*) for 3 hours at 37 °C. Following this treatment, 1–5 mL of each culture was centrifuged at 15,000 × g for 1 minute and washed twice with 1 mL of PBS (for *S. aureus*) or TBS (for *E. coli* and *K. pneumoniae*). The resulting *S. aureus* cell pellets were further processed and labeled with **BOFP** as described above for the Gram-positive bacterial strains and the resulting *E. coli* and *K. pneumoniae* cell pellets were further processed and labeled with **BOFP** as described above for the Gram-negative bacterial strains. The impact of treating *S. aureus* NRS705 cells with the FtsZ inhibitors **PC190723** and **TXA707** at 4× MIC (2 µg/mL for **PC190723** and 4 µg/mL for **TXA707**) was also examined using **BOFP** as described above.

To visualize the impact of treating MRSA LAC FtsZ-mCherry with **1**, cells were grown to log-phase in CAMH broth and diluted to an OD_600_ of 0.1. The cells were then treated with 10 µM IPTG and either DMSO vehicle or **1** at 4× MIC (0.25 µg/mL) for 3 hours at 37 °C. Following this treatment, 1–5 mL of each culture was centrifuged at 15,000 × g for 1 minute and washed twice with 1 mL of PBS. The resulting cell pellets were further processed and labeled with **BOFP** as described above for the Gram-positive bacterial strains.

## Supplementary information

Supplementary Information
