# Supplementary material for: Structure-Guided Design of a Fluorescent Probe for the Visualization of FtsZ in Clinically Important Gram-Positive and Gram-Negative Bacterial Pathogens
Source: Sci Rep. 2019 Dec 27;9:20092. doi: 10.1038/s41598-019-56557-x (PMC6934700; doi:10.1038/s41598-019-56557-x)
Supplement: Supplementary file 1 — Supplementary Information [file 41598_2019_56557_MOESM1_ESM.pdf]

Supplementary Information

Structure-Guided Design of a Fluorescent Probe for the Visualization of FtsZ in Clinically Important Gram-Positive and Gram-Negative Bacterial Pathogens

Edgar Ferrer-González[1], Junso Fujita[2,†], Takuya Yoshizawa[3], Julia M. Nelson[1], Alyssa J. Pilch[1], Elani Hillman[1], Mayuki Ozawa[3], Natsuko Kuroda[3], Hassan M. Al-Tameemi[4], Jeffrey M. Boyd[4], Edmond J. LaVoie[5], Hiroyoshi Matsumura[3,*], and Daniel S. Pilch[1,*]

[1]Department of Pharmacology, Rutgers Robert Wood Johnson Medical School, 675 Hoes Lane, Piscataway, NJ 08854, USA.

[2]Department of Applied Chemistry, Graduate School of Engineering, Osaka University, 2-1 Yamadaoka, Suita, Osaka 565-0871, JAPAN.

[3]Department of Biotechnology, College of Life Sciences, Ritsumeikan University, 1-1-1 Noji-Higashi, Shiga 525-8577, JAPAN.

[4]Department of Biochemistry and Microbiology, School of Environmental and Biological Sciences, Rutgers University, 76 Lipman Drive, New Brunswick, NJ 08901, USA.

[5]Department of Medicinal Chemistry, Ernest Mario School of Pharmacy, Rutgers University, 160 Frelinghuysen Road, Piscataway, NJ 08854, USA.

[†]Present address: MRC Laboratory of Molecular Biology, Francis Crick Avenue, Cambridge Biomedical Campus, Cambridge, CB2 0QH, UK.

*Correspondence and requests for materials should be addressed to D.S.P. (email: pilchds@rwjms.rutgers.edu) or H.M. (email: h-matsu@fc.ritsumei.ac.jp).

Supplemental Figures and Tables:

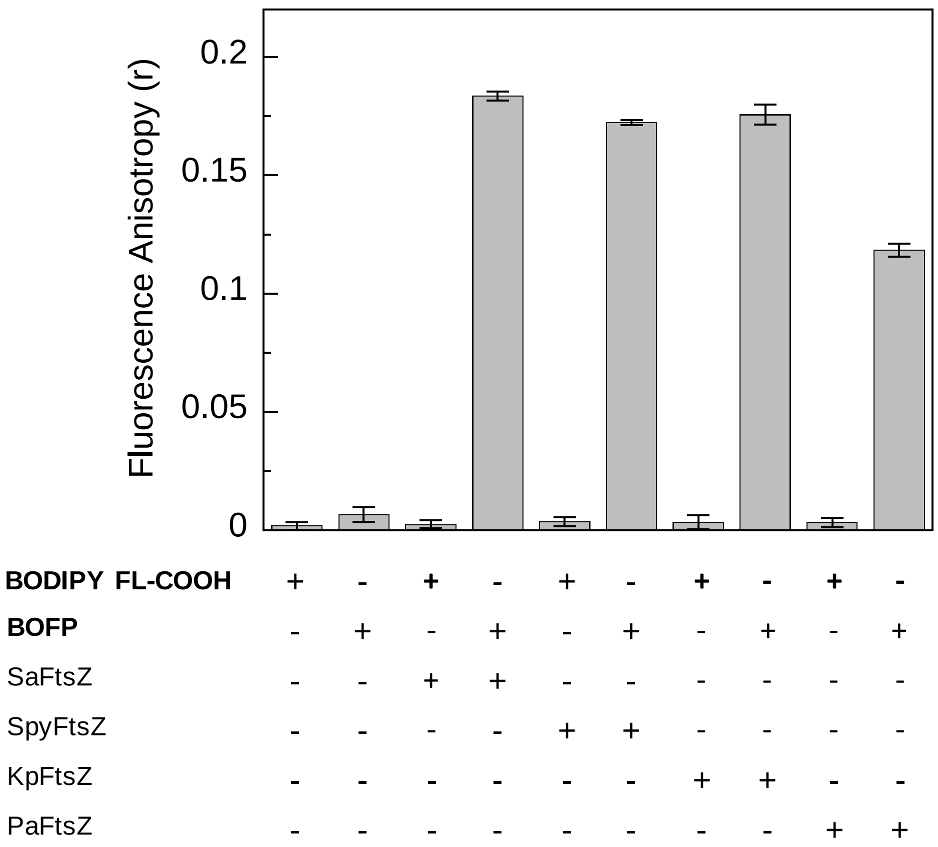

Figure S1. Fluorescence anisotropy of **BODIPY FL-COOH** (0.1 µM) or **BOFP** (0.1 µM) alone or in the presence of SaFtsZ (10 µM), SpyFtsZ (10 µM), KpFtsZ (2 µM), or PaFtsZ (2 µM). Anisotropy measurements were conducted at 37 °C in solution containing 50 mM Tris-HCl (pH 7.6) and 50 mM KCl.

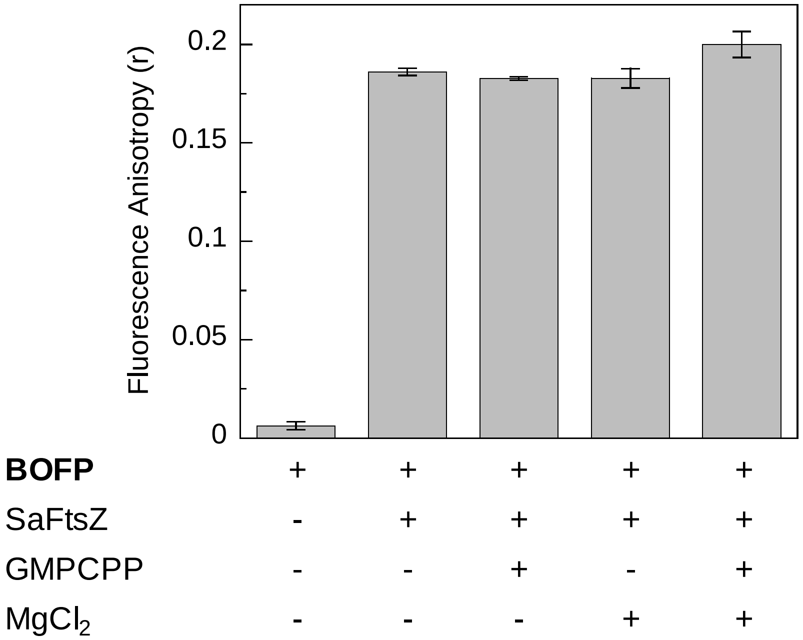

Figure S2. Fluorescence anisotropy of **BOFP** (0.1 μM) alone or in the presence of SaFtsZ (10 μM), with the latter also being shown in the presence of GMPCPP (0.1 mM), $MgCl_2$ (10 mM), or both. Anisotropy measurements were conducted at 37 °C in solution containing 50 mM Tris-HCl (pH 7.6) and 50 mM KCl.

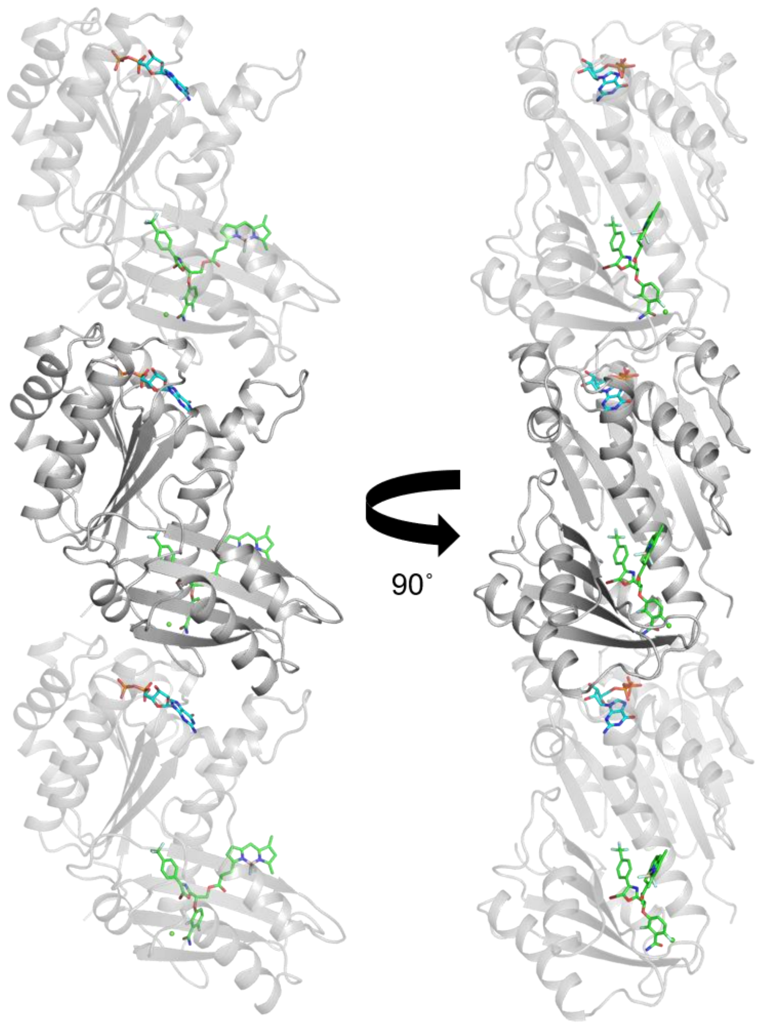

Figure S3. SaFtsZ forms a polymer in the crystal. In this trimeric representation, the R enantiomer of **BOFP** (green) and GDP (cyan) are depicted as stick models and the protein molecules (gray) are depicted as cartoon models.

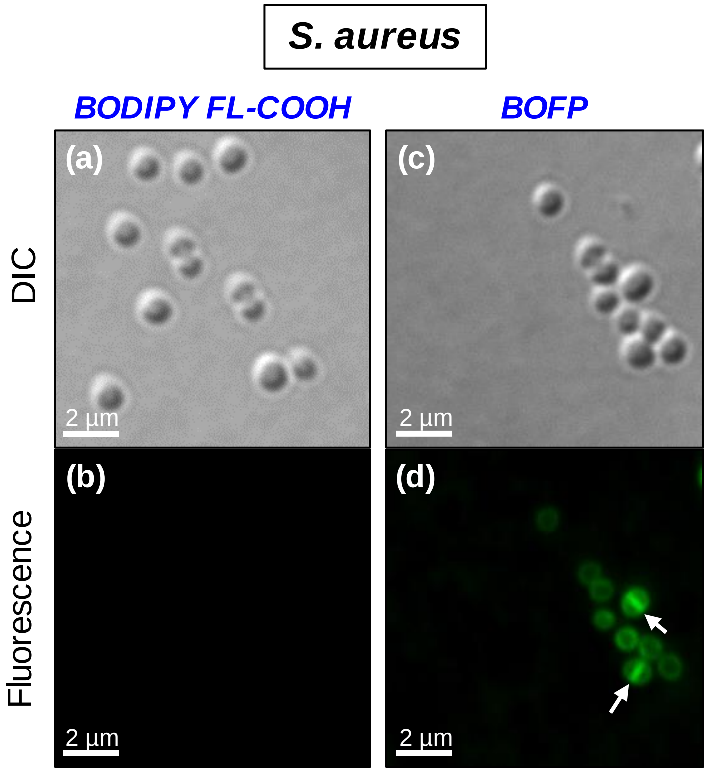

Figure S4. Comparison of **BODIPY FL-COOH** and **BOFP** for visualization of FtsZ in *S. aureus* NRS705 (MRSA). Differential interference contrast (DIC) and fluorescence micrographs of the bacteria treated for 5 minutes with 1 µg/mL **BODIPY FL-COOH** (a,b) or 1 µg/mL **BOFP** (c,d) just prior to visualization. The arrows in panel (d) highlight representative FtsZ Z-rings at midcell labeled by **BOFP**.

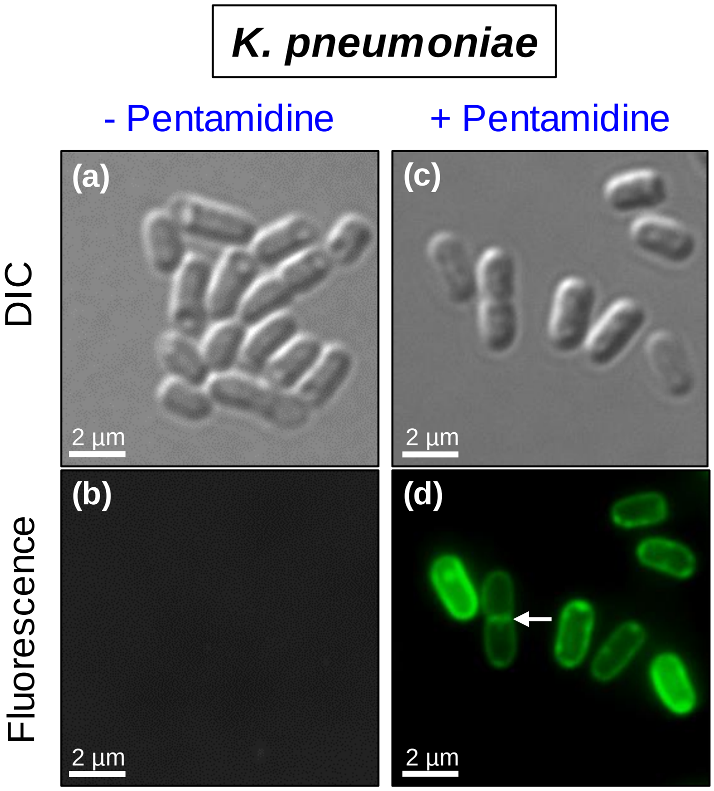

Figure S5. Impact of pentamidine isethionate on FtsZ visualization with **BOFP** in the Gram-negative bacterial pathogen *K. pneumoniae* ATCC 13883. Differential interference contrast (DIC) and fluorescence micrographs of the bacteria treated for 5 minutes with 1 µg/mL **BOFP** in the absence (a,b) or presence (c,d) of pentamidine isethionate (at 3.5 mg/mL) just prior to visualization. The arrow in panel (d) highlights a representative FtsZ Z-ring at midcell labeled by **BOFP**.

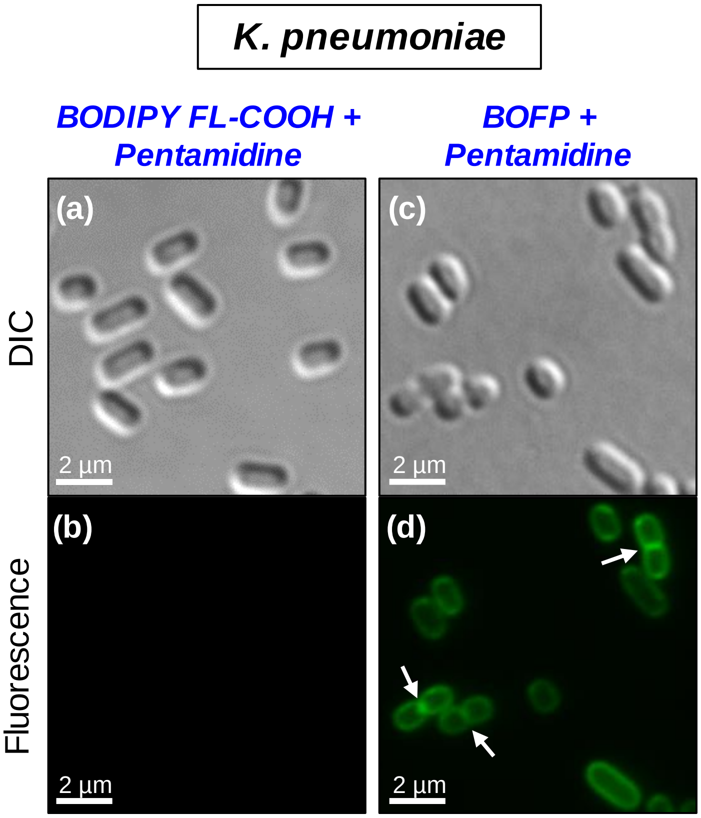

Figure S6. Comparison of **BODIPY FL-COOH** and **BOFP** for visualization of FtsZ in *K. pneumoniae* ATCC 13883. Differential interference contrast (DIC) and fluorescence micrographs of the bacteria treated for 5 minutes with 1 µg/mL **BODIPY FL-COOH** (a,b) or 1 µg/mL **BOFP** (c,d) in the presence of pentamidine isethionate (at 3.5 mg/mL) just prior to visualization. The arrows in panel (d) highlight representative FtsZ Z-rings at midcell labeled by **BOFP**.

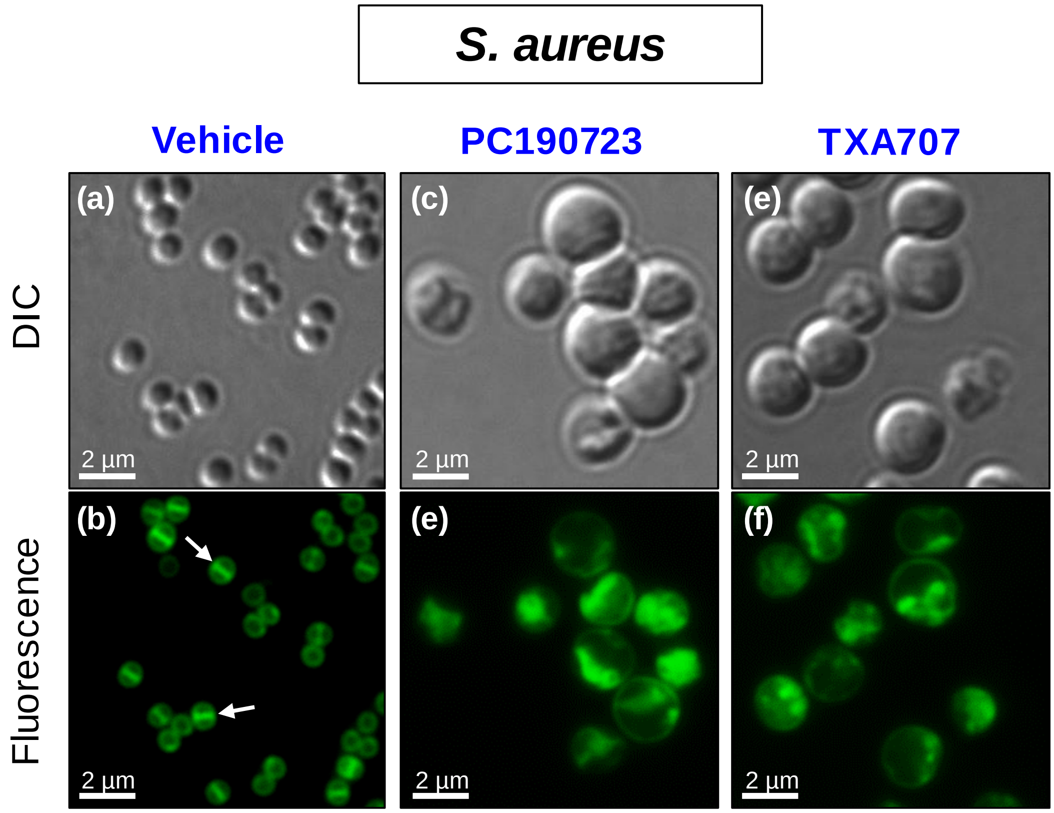

Figure S7. Visualization of the impact of treatment with **PC190723** or **TXA707** on FtsZ localization in *S. aureus* NRS705 (MRSA). Differential interference contrast (DIC) and fluorescence micrographs of the bacterial cells treated for 3 hours with DMSO vehicle (a,b), **PC190723** (c,d) at 4× MIC (2 µg/mL), or **TXA707** (e,f) at 4× MIC (4 µg/mL). Just prior to visualization, cells were labeled for 5 minutes with 1 µg/mL **BOFP**. The arrows in panel (b) highlight representative FtsZ Z-rings at midcell labeled by **BOFP**.

Name	*Sequence (5' → 3')
SaFtsZ-F	CTTTAAGAAGGAGATATACAATGTTAGAATTTGAACAAGGATTTAATC
SaFtsZ-R	GGTGCTCGAGTGCGGCCGCATTA**GTGGTGGTGGTGGTGGTG**ACGTCTTGTTCTTCTTGAAC
EfmFtsZ-F	CTTTAAGAAGGAGATATACAATGGAATTCTCAATTGATAATAATATC
EfmFtsZ-R	GGTGCTCGAGTGCGGCCGCATTA**GTGGTGGTGGTGGTGGTG**TTTTTTACGACGGAAAAAC
SpyFtsZ-F	CTTTAAGAAGGAGATATACATATGGCATTTTCATTTGATAC
SpyFtsZ-R	GGTGCTCGAGTGCGGCCGCAAGCTTA**GTGGTGGTGGTGGTGGTG**ACGGTTTTTAAAGAATGGAG
SagFtsZ-F	CTTTAAGAAGGAGATATACAATGGTATTTTCATTTGATACAGC
SagFtsZ-R	GGTGCTCGAGTGCGGCCGCATTA**GTGGTGGTGGTGGTGGTG**ACGGTTTTTAAAGAATGGA
KpFtsZ-F	CTTTAAGAAGGAGATATACATATGTTTGAACCTATGGAACTGAC
KpFtsZ-R	GGTGCTCGAGTGCGGCCGCAAGCTTA**GTGGTGGTGGTGGTGGTG**ATCGGCTTGCTTACGCAG
PaFtsZ-F	CTTTAAGAAGGAGATATACATATGTTTGAACTGGTCGATAACATCGC
PaFtsZ-R	GGTGCTCGAGTGCGGCCGCAAGCTTCA**GTGGTGGTGGTGGTGGTG**ATCGGCCTGACGACGCAG
AbFtsZ-F	CTTTAAGAAGGAGATATACATATGGCCTCATTTGAATTTATAG
AbFtsZ-R	GGTGCTCGAGTGCGGCCGCAAGCTTA**GTGGTGGTGGTGGTGGTG**CTTACGTTGCTGATTTTTC

Table S1. Sequences of the oligonucleotide primers used for cloning the FtsZ genes into the pET-22b(+) expression vector. *The underlined sequence reflects the vector complementary sequence, while the bold sequence encodes the 6x His-tag.

Name	*Sequence (5' → 3')
pCM11-LacI-F	AGTGCCAAGCTTCTGATATTCCATGCAAGCTAATTCCGG
pCM11-LacI-R	CACCATTAGGCACCCCAGGCTCACTGCCCGCTTTCCAG
pCM11-F	GCCTGGGGTGCCTAATG
pCM11-R	AATATCAGAAGCTTGGCACTG
pMUTIN-FtsZ-F	GGAGGTGATCTAGAGTCGAGGCCAATAAAACTAGGAGGAAATTTA
pMUTIN-FtsZ-R	TGCTCACCATGGAGGCGCCGCAGGAACGTCTTGTTCTTCTTGAA
mCherry-F	CGGCGCCTCCATGGTGAGCAAGGGCGAG
mCherry-R-pMUTIN	ATTAGGCGGGCTGCACTAGACTACTTGTACAGCTCGTCCATG
pMUTIN-F	TCTAGTGCAGCCCGCCTAATGAGCGGGCTTTTTTC
pMUTIN-R	CTCGACTCTAGATCACCTCCTTAAGCTTAA
pLL39-FtsZ-F	AGCTTAGATCTAATCGAATTGATATCCTAACAGCACAAGAGCG
pLL39-FtsZ-R	AAAAAGCTTGCATGCCTGCATCACTGCCCGCTTTCCAG
pLL39-F	TGCAGGCATGCAAGCTTTTTTATTACCTAC
pLL39-R	AATTCGATTAGATCTAAGCTTCGGGAA

Table S2. Sequences of the oligonucleotide primers used for the generation of the MRSA LAC strain expressing the FtsZ-mCherry fusion protein. *The underlined sequence reflects the 5 amino acid linker used to fuse FtsZ to mCherry.

Supplemental Methods:

General protocol for the cloning, expression, and purification of the FtsZ proteins from *S. aureus*, *E. faecium*, *S. pyogenes*, *S. agalactiae*, *K. pneumoniae*, *P. aeruginosa, and A. baumannii.* The genomic DNA for each organism was extracted from overnight cultures using the DNeasy UltraClean Microbial Kit (QIAGEN). The *ftsZ* gene for each organism was amplified from the genomic DNA using Q5 High-Fidelity DNA polymerase (New England Biolabs) and the appropriate primers (the sequences of which are listed in Table S1) designed to introduce a 6x His-tag at the *C*-terminus of each recombinant FtsZ protein. The expression vector pET-22b(+) (Novagen-EMD Chemicals) was linearized using the restriction enzymes *Nde*I and *Hind*III. The linearized plasmid and the amplified PCR products were combined and assembled using the NEBuilder HiFi DNA Assembly Cloning Kit (New England Biolabs), with the resulting recombinant plasmid being used to transform *E. coli* NEB5-α. The transformed *E. coli* NEB5-α were grown on Luria-Bertani (LB) agar plates containing 100 µg/mL ampicillin. Single colonies were selected for colony PCR to verify the presence of the proper insert in the pET-22b(+) plasmid. The recombinant plasmids were then isolated from *E. coli* NEB5-α and after verification of their sequences, were subsequently transformed into *E. coli* BLR (DE3) cells.

The transformed *E. coli* BLR (DE3) were grown on LB agar plates containing 100 µg/mL ampicillin. Single colonies were isolated and grown at 37 °C overnight in 50 mL of LB broth supplemented with 100 µg/mL ampicillin. The overnight cultures were diluted 1:100 into 2-4 L of LB broth containing 100 µg/mL ampicillin and incubated at 37 °C until the OD_{600} reached 0.6. The cultures expressing the FtsZ proteins from *S. agalactiae* and *E. faecium* were then induced with 1 mM isopropyl β-D-1-thiogalactopyranoside (IPTG) and incubated at 37 °C for an additional 5 hours. The cultures expressing all other FtsZ proteins were induced with 1 mM IPTG and incubated at 15 °C for 18 hours. The cells were harvested by centrifugation at 5,000 g for 15 minutes at 4 °C and the cell pellets were then resuspended in 10 mL of buffer A {50 mM Tris-HCl (pH 7.6), 300 mM NaCl, 20 mM imidazole} supplemented with 10% (v/v) glycerol and stored at -80 °C.

The cells were lysed by ultra-sonication (with a Qsonica Q500 sonicator equipped with a ½-inch probe) for 15 minutes at 0 °C, with an on/off cycle of 10 seconds at 60 W. The lysates were centrifuged at 10,000 g for 30 minutes at 4 °C and the resulting supernatants were added to 7 mL of TALON metal affinity resin (Clonetech Laboratories) and then shaken for 1 hour at 4 °C. The resin was then washed with 50 mL of buffer A and loaded into a gravity flow column. The protein was then eluted with 15 mL of elution buffer containing 50 mM Tris-HCl (pH 7.6), 300 mM NaCl, and 250 mM imidazole. Protein fractions were collected in volumes of 500 µL each. Each fraction was analyzed by SDS-PAGE and those containing the FtsZ protein of interest were combined. The combined fractions were dialyzed overnight at 4 °C in 4 L of buffer containing 50 mM Tris-HCl (pH 7.6) and 50 mM KCl, with the resulting dialysates being concentrated to a volume of 0.2-1 mL using Amicon Ultra-4 10K filters (EMD Millipore). Protein concentrations were determined using the Pierce BCA Protein Assay Kit (ThermoFisher). The protein solutions were then aliquoted, snap-frozen in liquid nitrogen, and stored at -80 °C.

Generation of a MRSA LAC strain expressing an inducible FtsZ-mCherry fusion protein. We constructed a MRSA strain in which a FtsZ-mCherry fusion protein is ectopically expressed under control of the isopropyl β-d-1-thiogalactopyranoside (IPTG)-inducible P*spac* promoter. A list of primers required for the amplification of each DNA fragment is listed on Table S2. In order to

control the expression of FtsZ-mCherry, we introduced a multicopy plasmid constitutively expressing the LacI repressor. For generation of this initial plasmid, we first amplified the *lac*I repressor gene from the pMUTIN-HA[1] plasmid using Q5 High-Fidelity DNA polymerase and the pCM11-LacI-F and pCM11-LacI-R primers. The multicopy vector pCM11[2] was then amplified using Q5 High-Fidelity DNA polymerase and the pCM11-F and pCM11-R primers. This process resulted in the deletion of the *lac*O sequence. The amplicons resulting from the two amplification reactions described above were then combined and assembled using the NEBuilder HiFi DNA Assembly Cloning Kit to form the plasmid pCM11-*lac*I. pCM11-*lac*I was then transformed into *E. coli* NEB5-α and its sequence subsequently verified. pCM11-*lac*I was then electroporated into *S. aureus* RN4220 (an MSSA strain) and subsequently transduced into *S. aureus* LAC (a USA300 MRSA strain) using the bacteriophage 80α[3], thereby resulting in the strain MRSA LAC *lac*I.

We then proceeded to generate the fusion protein FtsZ-mCherry by amplifying the FtsZ gene from the MRSA LAC genome using the pMUTIN-FtsZ-F and pMUTIN-FtsZ-R primers. The mCherry reporter gene was amplified from the plasmid pmCherry (Clonetech Laboratories) using the mCherry-F and mCherry-R-pMUTIN primers. The pMUTIN-HA plasmid was then amplified using the pMUTIN-F and pMUTIN-R primers, which resulted in the deletion of 10 nucleotides in the multiple cloning site. The amplicons resulting from the three amplification reactions described above were then combined and assembled using the NEBuilder HiFi DNA Assembly Cloning Kit to form the plasmid pMUTIN-FtsZ-mCherry, which now contains the gene encoding the FtsZ-mCherry fusion protein under control of the P*spac* promoter. pMUTIN-FtsZ-mCherry was then transformed into *E. coli* NEB5-α and its sequence subsequently verified.

To integrate the gene encoding the FtsZ-mCherry fusion protein into the chromosome of MRSA LAC we amplified the DNA sequence containing the P*spac* promoter and the *fts*Z-*mCherry* gene from the pMUTIN-FtsZ-mCherry plasmid using the pLL39-FtsZ-F and pLL39-FtsZ-R primers. The single-copy integration vector pLL39[4] was then amplified using the pLL39-F and pLL39-R primers. The amplicons from the two amplification reactions described above were then combined and assembled using the NEBuilder HiFi DNA Assembly Cloning Kit to form the pLL39-FtsZ-mCherry plasmid. pLL39-FtsZ-mCherry was then transformed into *E. coli* NEB5-α and its sequence verified. pLL39-FtsZ-mCherry was then electroporated into an MSSA RN4220 strain containing the pLL2787 plasmid that expresses the ϕ11 *int* gene[4], which in turn resulted in the integration of the DNA sequence containing the P*spac* promoter and the *fts*Z-*mCherry* gene at the ϕ11 attB site of the bacterial chromosome. The resulting genome was subsequently transduced into the MRSA LAC *lac*I strain using the bacteriophage 80α, resulting in generation of the MRSA LAC FtsZ-mCherry strain. The presence of the DNA sequence containing the P*spac* promoter and the *fts*Z-*mCherry* gene in the genome of MRSA LAC FtsZ-mCherry was confirmed by PCR.

Synthesis of BOFP. As schematically depicted in Fig. 2, 2-(5-bromo-4-(4-(trifluoromethyl)phenyl)oxazol-2-yl)-2-(3-carbamoyl-2,4-difluorophenoxy)ethyl 3-(5,5-difluoro-7,9-dimethyl-5*H*-5λ^4,6λ^4-dipyrrolo[1,2-*c*:2',1'-*f*][1,3,2]diazaborinin-3-yl)propanoate (**BOFP**) was synthesized by adding **BODIPY FL-COOH** (12 mg, 0.04 mmol), 1-ethyl-3-(3-dimethylaminopropyl)carbodiimide (EDC) (12 mg, 0.06 mmol), and 4-dimethylaminopyridine (DMAP) (5.0 mg, 0.04 mmol) to **3** (20 mg, 0.04 mmol) in CH_2Cl_2 (3 mL). The reaction mixture was stirred at room temperature overnight. The reaction mixture was then diluted with CH_2Cl_2 and washed with brine. The organic layer was dried over sodium sulfate and filtered. The filtrate was then concentrated and purified using ISCO column chromatography on silica gel (50% ethyl acetate/hexanes) to give the product (25 mg, 82% yield) as a deep red solid. ^{1}H NMR (300 MHz,

$CDCl_3$) δ: 8.06 (d, *J* = 8.41 Hz, 1H), 7.68 (d, *J* = 9.0 Hz, 1H), 7.19 (m, 1H), 6.83 (m, 1H), 6.81 (d, *J* = 3.9 Hz, 1H), 6.23 (d, *J* = 3.9 Hz, 1H), 6.11 (s, 2H), 5.86 (s, 2H), 5.42 (dd, *J* = 4.8, 7.8 Hz, 1H), 4.81 (dd, *J* = 7.8, 11.4 Hz, 1H), 4.67 (dd, *J* = 4.8, 11.7 Hz, 1H), 3.27 (t, *J* = 7.8 Hz, 1H), 2.81 (t, *J* = 7.5 Hz, 1H), 2.53 (s, 3H), 2.24 (s, 3H). ^{13}C NMR (400 MHz, $CDCl_3$) δ: 171.91, 161.42, 160.70, 159.70, 156.29, 144.22, 136.49, 135.31, 133.24, 132.81, 127.97, 126.71, 125.58, 125.53, 123.87, 121.68, 120.62, 119.72, 116.49, 111.82, 111.58, 74.37, 63.47, 33.16, 23.81, 14.92, 11.28. Electrospray ionization (ESI) high-resolution mass spectrometry (HRMS) of BODIPY dyes typically yields $[M+H-F]^+$ as the most abundant product ion, due to neutral loss of F[5]. We also observed $[M+H-F]^+$ as the most abundant ion of our reaction product, with the ESI HRMS of $C_{33}H_{25}BBrF_7N_4O_5$ being as follows: $[M+H-F]^+$ calculated 761.1006, found 761.1025.

Supplemental References:

1. Kaltwasser, M., Wiegert, T. & Schumann, W. Construction and application of epitope- and green fluorescent protein-tagging integration vectors for *Bacillus subtilis*. *Appl. Environ. Microbiol.* **68**, 2624-2628 (2002).
2. Malone, C. L. *et al.* Fluorescent Reporters for *Staphylococcus aureus*. *J. Microbiol. Methods* **77**, 251-260 (2009).
3. Novick, R. P. Genetic systems in staphylococci. *Methods Enzymol.* **204**, 587-636 (1991).
4. Luong, T. T. & Lee, C. Y. Improved single-copy integration vectors for *Staphylococcus aureus*. *J. Microbiol. Methods* **70**, 186-190 (2007).
5. Qi, Y., Geib, T., Huynh, A.-M., Jung, G. & Volmer, D. A. Fragmentation Patterns of Boron-Dipyrromethene (BODIPY) Dyes by Electrospray Ionization High-Resolution Tandem Mass Spectrometry. *Rapid. Commun. Mass. Spectrom.* **29**, 885-890 (2015).
